# An Assisting Contact Electrification Strategy for Achieving Self‐Recoverable Mechanoluminescence

**DOI:** 10.1002/advs.76254

**Published:** 2026-06-30

**Authors:** Jianwen Zhang, Weiguang Wang, Jinyu Zhou, Jiachi Zhang, Wenxiang Wang, Shanwen Wang, Jing Liu, Haoyang Li, Xianfeng Jin, Chi Zhang, Ziyuan Li, Zhaofeng Wang, Yuhua Wang

**Affiliations:** ^1^ National & Local Joint Engineering Laboratory for Optical Conversion Materials and Technology Lanzhou University Lanzhou P. R. China; ^2^ School of Materials Science and Engineering Key Laboratory of Anisotropy and Texture of Materials (Ministry of Education) Northeastern University Shenyang P. R. China; ^3^ Shandong Laboratory of Advanced Materials and Green Manufacturing At Yantai Yantai P. R. China; ^4^ State Key Laboratory of Solid Lubrication Lanzhou Institute of Chemical Physics Chinese Academy of Sciences Lanzhou P. R. China

**Keywords:** contact electrification, LaPO_4_: Tb^3+^, Ce^3+^, phosphor, self‐recoverable mechanoluminescence

## Abstract

Self‐recoverable mechanoluminescence (ML) is a unique property of materials that can stably emit light under repeated stress, enabling new applications in smart sensing, displays, and wearable devices. However, self‐recoverable ML is very rare and only observed in a few active phosphors excited by piezoelectricity or contact electrification. Herein, we present an assisting contact electrification strategy that can enable inactive phosphors inherently lacking ML to achieve self‐recoverable ML. A typical illustration demonstrates that commercially available LaPO_4_: Tb^3+^, Ce^3+^ phosphor lacks piezoelectrically excited ML. No ML of Tb^3+^ is excited in polydimethylsiloxane (PDMS), apart from the ML of LaPO_4_ host. Accordingly, we introduce strongly electrifiable fluoride into PDMS to induce contact electrification, thereby successfully assisting in the excitation of self‐recoverable ML of Tb^3+^ from the inactive LaPO_4_: Tb^3+^, Ce^3+^. This case illustrates that, based on the assisting contact electrification strategy, the originally rare self‐recoverable ML may become a universal property of all phosphors.

## Abstract 2

This graphic draws inspiration from Chinese mythology. By employing the assisting contact electrification strategy, the Monkey King (Sun Wukong) and the Celestial Master Laozi enabled the non‐ML‐active divine stone to exhibit highly efficient self‐recoverable ML. This graphic vividly summarizes our core theme: “Man conquers nature.”

## 1 Introduction

Mechanoluminescence (ML) refers to the phenomenon where certain materials emit light when subjected to external mechanical stresses such as friction, stretching, compression, or vibration [1, 2, 3]. If the ML intensity of a material can spontaneously recover upon each stress application, it is defined as self‐recoverable ML [4, 5, 6]. Excited directly by stress, self‐recoverable ML requires no irradiation source or power supply, enabling light emission under any conditions [7, 8, 9, 10]. Moreover, the ML intensity depends linearly on the applied magnitude of stress, so the ML brightness can reflect the local stress distribution across the material [11, 12, 13]. Owing to these unique mechano‐optical conversion characteristics, self‐recoverable ML materials have attracted extensive attention in recent years and are actively being studied for applications in smart sensing, anti‐counterfeiting, displays, and wearable devices [14, 15, 16, 17, 18, 19].

Despite their promising application prospects, practically useful self‐recoverable ML materials remain exceedingly rare [20, 21]. As early as 1605, the ML phenomenon was first discovered by the famous scientist Bacon in an experiment of scratching sugar, which was recorded in his work “Advancement of Learning” [22, 23]. However, this bond‐breaking‐induced ML is one‐time and non‐recoverable, thus lacking practical application value [24, 25, 26]. Over the following centuries, ML was subsequently observed in dozens of piezoelectric materials; however, to date, only a few sulfides and oxysulfides have shown piezoelectricity‐excited self‐recoverable ML [27, 28]. In recent years, a few phosphor/polydimethylsiloxane (PDMS) composites were demonstrated to show self‐recoverable ML due to contact electrification [29, 30, 31, 32]. Nevertheless, to meet the requirements of practical applications, there is an urgent need to develop more stable, efficient, and multicolor self‐recoverable ML materials. In particular, if existing commercial lighting or display phosphors could emit self‐recoverable ML, it would greatly advance the ML applications. Unfortunately, most commercial phosphors, particularly oxides known for their high luminescence efficiency, have been confirmed to lack self‐recoverable ML, which severely limits the development and application of ML technology [33, 34, 35]. More recently, researchers have discovered that some fluoride phosphors can emit self‐recoverable ML in PDMS [36, 37, 38]. Our research revealed that fluoride surfaces have low contact state energy, enabling them to induce strong contact electrification effects in PDMS, thereby exciting self‐recoverable ML [39, 40, 41]. Regrettably, most fluoride phosphors inherently suffer from low luminescence efficiency [42, 43]. Consequently, despite inducing strong contact electrification in PDMS, the excited self‐recoverable ML brightness of fluoride phosphors remains weak. This situation creates a performance mismatch: oxides have high luminescence efficiency but weak contact electrification capability, making them unable to excite self‐recoverable ML. Conversely, fluorides have strong contact electrification capability to excite self‐recoverable ML, but they cannot emit light efficiently themselves. This raises a key question: Is there a method to combine the high luminescence efficiency of oxides with the strong contact electrification capability of fluorides to achieve high‐brightness self‐recoverable ML? Based on the principle of contact electrification, we designed an assisting contact electrification strategy: fabricating a “fluoride + oxide/PDMS” composite material. The concept utilizes the strongly electrifiable fluoride to interact with PDMS instead of the weakly electrifiable oxide phosphor, thereby inducing strong contact electrification and assist in exciting the self‐recoverable ML of high‐efficiency oxide phosphor. Is this assisting contact electrification strategy truly effective? To address this question, experimental validation is required.

In this work, we selected the typical commercial lighting oxide phosphor LaPO_4_: Tb^3+^, Ce^3+^ as a demonstration case and employed the strongly electrifiable fluoride AlF_3_ as an assisting material. Using simple processes of physical mixing and mixed sintering, we prepared AlF_3_‐LaPO_4_: Tb^3+^, Ce^3+^/PDMS composites. The results indicate that the LaPO_4_: Tb^3+^, Ce^3+^ phosphor exhibits neither piezoelectricity nor contact electrification, excited self‐recoverable ML of Tb^3+^. However, when an appropriate amount of AlF_3_ is introduced into the PDMS as an assisting contact electrification material, bright green self‐recoverable ML of the LaPO_4_: Tb^3+^, Ce^3+^ was clearly observed. This case verifies the effectiveness of the proposed assisting contact electrification strategy. It also suggests that, based on this strategy, the originally rare self‐recoverable ML may become a universal property of all phosphors.

## 2 Results and Discussion

**FIGURE 1:**
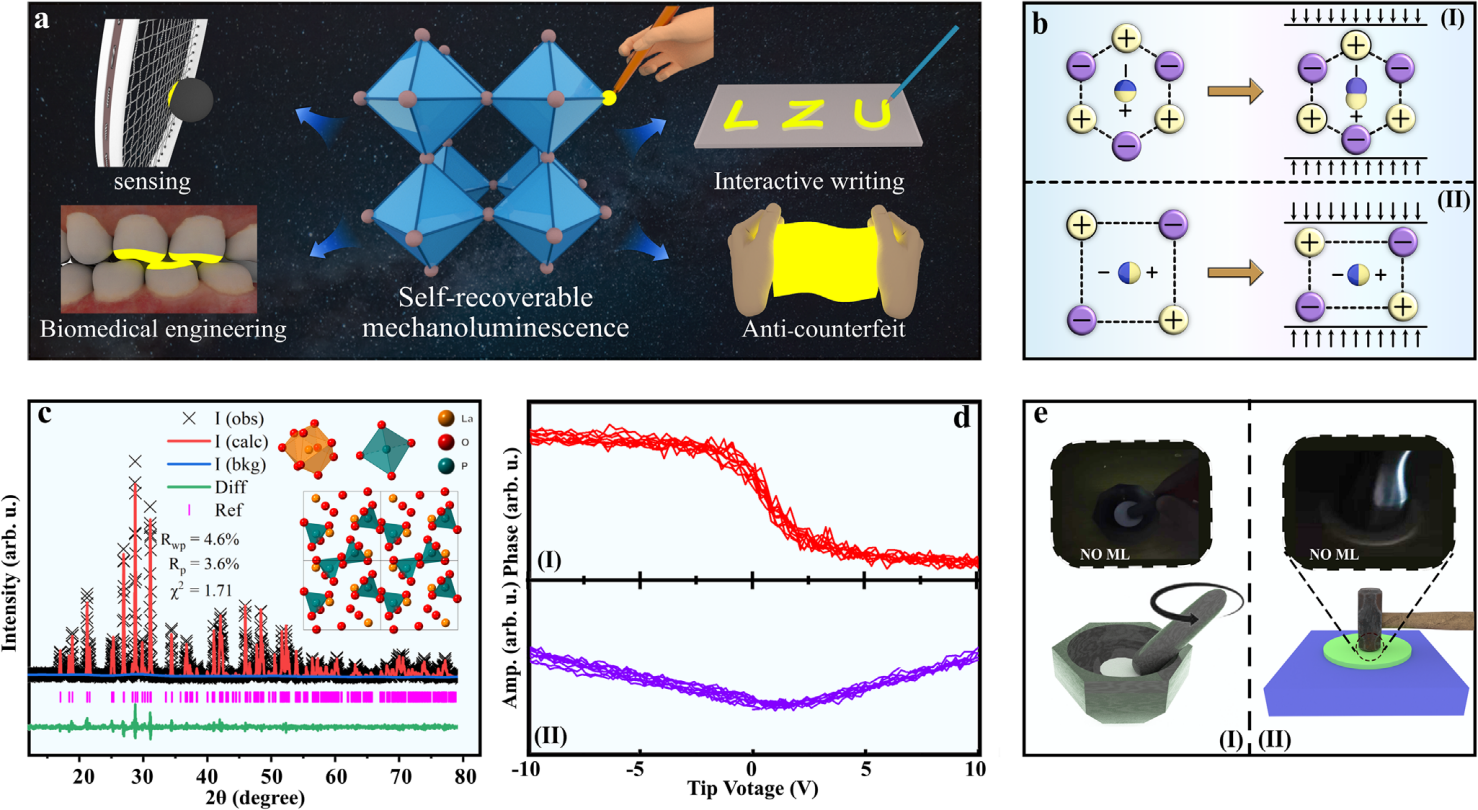
The concept of self‐recoverable ML and the piezoelectricity‐excited ML performance (a) Concept of self‐recoverable ML and its wide applications; (b) Changes in charge centers of asymmetric piezoelectric structure (I) and centrosymmetric structure (II) crystals under stress; (c) Rietveld refinement results of LaPO_4_: Tb^3+^, Ce^3+^ phosphor, as well as the centrosymmetric unit cell and cationic polyhedrons of LaPO_4_ crystal; (d) Phase (I) and amplitude (II) of piezo‐response force microscopies of LaPO_4_; (e) Schematic diagrams: grinding LaPO_4_: Tb^3+^, Ce^3+^ phosphor in a mortar (I) and striking LaPO_4_: Tb^3+^, Ce^3+^/epoxy‐resin discs with a hammer (II) in a dark environment, and no ML was observed in either case (insets).

First, it is essential to determine whether LaPO_4_: Tb^3+^, Ce^3+^ shows ML of Tb^3+^. Generally, the self‐recoverable ML of phosphor can be excited by piezoelectricity or by contact electrification [44, 45, 46, 47]. As shown in Figure 1b‐I, when asymmetric piezoelectric materials are subjected to external stress, the crystal lattice undergoes a certain structural separation that generates piezoelectricity, which may excite the self‐recoverable ML of the phosphor [48, 49, 50]. However, when the crystal structure is centrosymmetric, no structural strain can result in charge separation (Figure 1b‐II), and thus no piezoelectricity is generated. Therefore, phosphors with centrosymmetric structures can only exhibit ML through the contact electrification effect [51, 52, 53].

Next, we need to verify whether LaPO_4_: Tb^3+^, Ce^3+^ has piezoelectrically excited self‐recoverable ML. Figure 1c and Table S1 show the Rietveld refinement results of the LaPO_4_: Tb^3+^, Ce^3+^. The results indicate: the LaPO_4_ crystal belongs to a typical monoclinic system with a space group of P21/c (No. 14); as shown in the inset of Figure 1c, the unit cell of the LaPO_4_ is invariant under a 180° rotation about its center, indicating a centrosymmetric structure (i.e., nonpiezoelectric structure) [54, 55, 56]. Additionally, the electronic structure of LaPO_4_ (Figures S1–S5) shows that LaPO_4_ has an indirect band gap with a width as large as 4.82 eV, implying low electrical conductivity and difficulty in forming a stable piezoelectric response [57, 58, 59]. These results theoretically suggest that the LaPO_4_: Tb^3+^, Ce^3+^ phosphor is unlikely to emit piezoelectrically excited self‐recoverable ML. Nevertheless, previous literature reports suggest that trace doping may destroy the local symmetry of centrosymmetric crystals, potentially inducing local piezoelectric effects [60, 61, 62, 63]. In this case, the LaPO_4_ crystal mainly contains two cation‐centered polyhedrons: PO_4_ tetrahedrons and LaO_9_ decahedrons (Figure 1c and Table S2). According to Hume‐Rothery rules (Tables S3–S5), the ionic radius difference and electronegativity difference between Ce^3+^ and La^3+^ are merely 1.57% and 1.20%, respectively. For Tb^3+^ and La^3+^, the corresponding differences are 18.11% and 5.12%. Both dopants exhibit excellent size compatibility and chemical affinity with La^3+^. Therefore, the doped Ce^3+^ and Tb^3+^ ions preferentially replace the La^3+^ lattice sites at the centers of LaO_9_ decahedrons [64, 65]. Elemental mapping of the LaPO_4_: Tb^3+^, Ce^3+^ sample particles (Figures S6–S11) confirms the presence of the doped Ce and Tb elements, while x‐ray photoelectron spectroscopy (Figures S12–S14) further confirms that both Ce and Tb dopants are in the +3 valence state. Generally, the inherent differences in ionic radius and coordination environment among Ce^3+^, Tb^3+^, and La^3+^ will induce distortion of local La‐O bond lengths and bond angles after doping, breaking the original centrosymmetric lattice symmetry around doping sites and thereby causing a weak local polarization effect. Accordingly, Figure 1d shows the piezo‐response force microscopies of the LaPO_4_: Tb^3+^, Ce^3+^ samples. We can clearly observe overlapping hysteresis loops characteristics of non‐piezoelectric behavior. This result demonstrates experimentally that trace doping with Ce^3+^ and Tb^3+^ does not disrupt the global or local centrosymmetry of the crystal lattice, and thus no local piezoelectricity is induced [66, 67, 68]. Finally, as shown in Figure 1e‐I, direct grinding of LaPO_4_: Tb^3+^, Ce^3+^ powder in the dark yielded no detectable ML emission. Similarly, when the phosphor was embedded in epoxy resin to form rigid discs and mechanically impacted with a hammer, no ML was observed as well (Figure 1e‐II). These experimental observations conclusively confirm the absence of piezoelectrically excited self‐recoverable ML in the LaPO_4_: Tb^3+^, Ce^3+^ phosphor.

**FIGURE 2:**
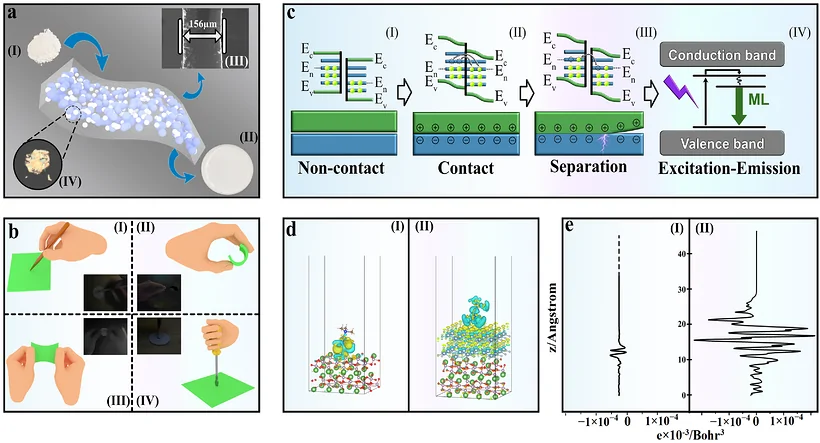
Contact electrification‐excited self‐recoverable ML mechanism and phosphor/PDMS model calculations. (a) Schematic diagrams: the fabrication of LaPO_4_: Tb^3+^, Ce^3+^/PDMS composite elastomer, as well as photos of LaPO_4_: Tb^3+^, Ce^3+^ powder (I), composite elastomer (II), scanning electron microscope image of the cross‐section of the composite elastomer (III), and optical microscope image of LaPO_4_: Tb^3+^, Ce^3+^ particles in the composite elastomer (IV); (b) Schematic diagrams: actions and ML photos of the composite elastomer under scratching (I), folding (II), stretching (III), and pressing (IV); (c) Contact electrification effect of phosphor and PDMS during non‐contact (I), contact (II), and separation (III) processes, as well as the mechanism of contact electrification‐induced self‐recoverable ML of phosphor/PDMS (IV); (d) Contact interface models of LaPO_4_ /PDMS (I) and AlF_3_&LaPO_4_/PDMS (II); (e) Calculated differential charge densities of LaPO_4_/PDMS (I) and AlF_3_&LaPO_4_/PDMS (II) models.

Subsequently, we aim to verify whether the LaPO_4_: Tb^3+^, Ce^3+^ exhibits contact electrification‐excited self‐recoverable ML of Tb^3+^ in PDMS. Accordingly, the LaPO_4_: Tb^3+^, Ce^3+^ particles (Figure 2a‐I) were incorporated into PDMS to fabricate a LaPO_4_: Tb^3+^, Ce^3+^/PDMS composite elastomer (Figure 2a‐II). The thickness of the obtained composite elastomer is about 156 µm (Figure 2a‐III), and the LaPO_4_: Tb^3+^, Ce^3+^ particles uniformly dispersed throughout the composite elastomer (Figure 2a‐IV). Then, various mechanical stimuli were manually applied to the elastomer in the dark, including: (1) scratching the elastomer surface with a metal rod (Figure 2b‐I); (2) folding the elastomer from the middle (Figure 2b‐II); (3) stretching the elastomer from both ends (Figure 2b‐III); (4) pressing the elastomer with a glass rod (Figure 2b‐IV). No characteristic green ML of Tb^3+^ was observed in all manual tests, even though LaPO_4_ shows a high relative work function in PDMS. This suggests that contact electrification depends on multiple factors beyond the work function alone. Moreover, upon application of high‐frequency stress via a friction tester, the LaPO_4_: Tb^3+^, Ce^3+^/PDMS composite shows intrinsic bluish‐violet ML peaking at 400 nm due to the LaPO_4_ host. Such intrinsic ML may arise from high‐energy excitations induced by the fracture of PDMS under high‐speed friction, and its properties and mechanism will be elaborated in a separate study.

These results collectively demonstrate that the LaPO_4_: Tb^3+^, Ce^3+^ phosphor intrinsically lacks piezoelectricity and is unable to generate an effective contact electrification effect when embedded in PDMS. Consequently, it cannot exhibit self‐recoverable ML of Tb^3+^. Can we enable the “useless” LaPO_4_: Tb^3+^, Ce^3+^ phosphor to emit self‐recoverable ML of Tb^3+^? At this stage, converting the inherently centrosymmetric LaPO_4_ crystal into a non‐centrosymmetric piezoelectric structure would be fundamentally contradictory. However, introducing a third component into the PDMS to efficiently induce contact electrification is theoretically feasible. According to previous studies, the physical process of contact electrification‐excited ML can be described as shown in Figure 2c [69, 70]. After the phosphor is compounded with the PDMS matrix, intimate interfacial contact forms between them. Driven by the contact electrification effect, electrons transfer from the high potential well to the low potential well until interfacial charge equilibrium is established. Upon application of external stress, mechanical friction causes rapid separation of the phosphor and PDMS surfaces. Simultaneously, the residual electrons and holes on the interfaces construct a strong excitation electric field, thereby exciting the luminescent center [71, 72, 73]. As for the electron transfer direction, we notice that most existing studies hold the view that electrons transfer from phosphors to PDMS upon contact. However, some of our authors hold a different view. At this stage, we decided not to describe the direction of electron transfer when the phosphor and PDMS come into contact. Instead, in Note S1 we explain why some of us are more inclined to believe that electrons transfer from PDMS to the phosphor surface for academic discussion and reference. According to this ML mechanism, for a phosphor/PDMS composite, contact electrification originates from the potential well energy level difference between the phosphor and PDMS [74, 75]. Significantly, a larger energy difference leads to greater charge transfer and thus stronger contact electrification capability. To quantitatively assess this capability, we constructed a computational model of the phosphor/PDMS interface and performed first‐principles calculations to determine the differential charge density upon contact. This parameter provides a direct measure of a phosphor's ability to induce contact electrification within PDMS (details regarding model construction and calculation methodology are provided in Figures S15–S22). As a typical example, Figure 2d compares the interface models of LaPO_4_/PDMS and AlF_3_&LaPO_4_/PDMS. Figure 2e displays the calculated differential charge densities of the two models. The results reveal a significant contrast: the differential charge density for the LaPO_4_/PDMS model is merely ±0.2 × 10^−4^ e/Bohr^3^, significantly lower than that calculated for the AlF_3_&LaPO_4_/PDMS model (±2.5 × 10^−4^ e/Bohr^3^). This theoretical finding provides direct evidence supporting the hypothesis that oxide LaPO_4_ possesses a weak contact electrification capability within PDMS, whereas fluoride AlF_3_ exhibits a remarkably strong capability. This conclusion aligns perfectly with our previous experimental observations [76, 77, 78].

**FIGURE 3:**
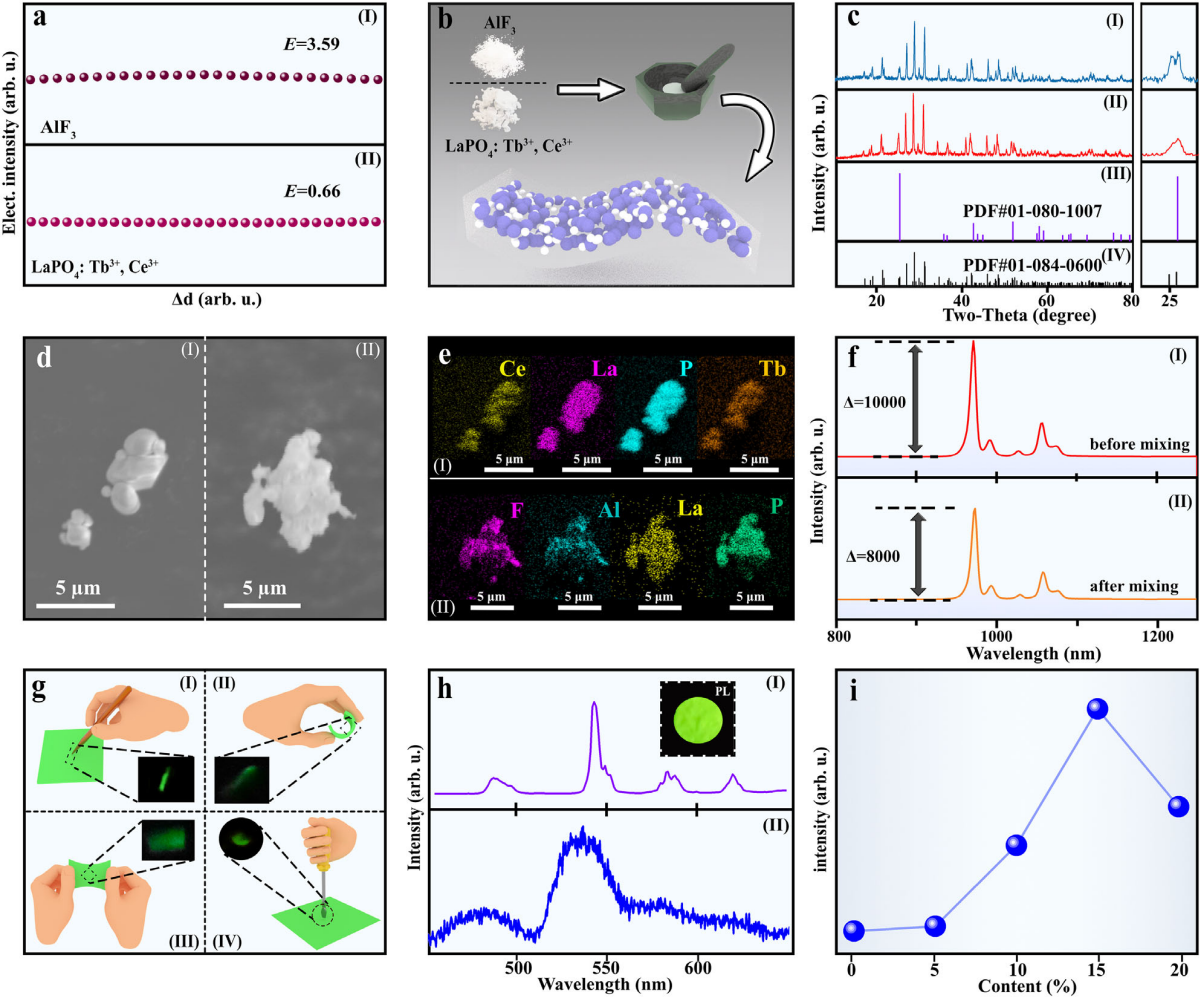
Enhancement of self‐recoverable ML. (a) Excitation electric field strengths on the surfaces of AlF_3_ (I) and LaPO_4_: Tb^3+^, Ce^3+^ phosphor (II) particles; (b) Schematic diagrams: physically mixing an appropriate amount of AlF_3_ and LaPO_4_: Tb^3+^, Ce^3+^ phosphor to prepare a composite elastomer; (c) XRD patterns of samples before (I) and after (II) mixing with AlF_3_, as well as standard x‐ray diffraction lines of AlF_3_ (III) and LaPO_4_ (IV); (d) Scanning electron microscope images of samples before (I) and after (II) mixing with AlF_3_; (e) Elemental distribution maps of samples before (I) and after (II) mixing with AlF_3_; (f) Raman spectra of samples before (I) and after (II) mixing with AlF_3_; (g) Schematic diagrams: actions and ML photos of AlF_3_&LaPO_4_: Tb^3+^, Ce^3+^/PDMS composite elastomer under scratching (I), folding (II), stretching (III), and pressing (IV); (h) PL spectrum and photo (I) of AlF_3_&LaPO_4_: Tb^3+^, Ce^3+^/PDMS composite elastomer, as well as its ML spectrum (II); (i) Relationship between the mass ratio of AlF_3_ and the self‐recoverable ML brightness of the composite elastomer.

Based on the above results, we propose an “assisting contact electrification” strategy, which involves introducing a fluoride with strong contact electrification capability into the oxide‐phosphor/PDMS composite. The fluoride induces a strong contact‐electrification effect within the PDMS, thereby assisting in exciting the self‐recoverable ML of the highly efficient oxide phosphor. In this case, aluminum fluoride (AlF_3_) was selected as the assisting material. AlF_3_ not only has a large theoretical differential charge density, but also generates an excitation electric field strength of 3.59, significantly higher than that of the LaPO_4_: Tb^3+^, Ce^3+^ (0.66), as shown in Figure 3a and Figure S23 [79, 80]. Accordingly, Figure 3b shows that the LaPO_4_: Tb^3+^, Ce^3+^ was uniformly mixed with varying amounts of AlF_3_ (mass ratio ranging from 5% to 20%) in a mortar. In this work, all mass ratios of AlF_3_ are calculated relative to the mass of LaPO_4_:Tb^3+^,Ce^3+^ phosphor. Figure 3c shows the XRD patterns of the LaPO_4_: Tb^3+^, Ce^3+^ before and after mixing with AlF_3_. The pattern shows that the primary phase in the mixture is LaPO_4_, alongside observable characteristic diffraction peaks of AlF_3_. SEM images in Figure 3d show that before mixing with AlF_3_, the surface of the LaPO_4_: Tb^3+^, Ce^3+^ particles is smooth, and the particle outline can be clearly seen (I); but after mixing with AlF3, many fine AlF_3_ particles adhere to the surface, making it almost impossible to see the surface and particle outline of the LaPO_4_: Tb^3+^, Ce^3+^ (II). Elemental mapping in Figure 3e further demonstrates that the distributions of La and P elements are similar to those of Al and F elements, although the latter exhibit a broader distribution range. This result also indicates that the AlF_3_ is uniformly dispersed on the surface of the LaPO_4_: Tb^3+^, Ce^3+^ particles. Raman spectra in Figure 3f show no significant changes in the characteristic peaks of the LaPO_4_ crystal before and after mixing with AlF_3_. This suggests that the addition of the AlF_3_ did not markedly affect the surface lattice periodicity of LaPO_4_. Therefore, the interface between AlF_3_ and LaPO_4_ remains physically weak, suggesting minimal chemical interaction or tight bonding in the mixture.

Subsequently, the obtained AlF_3_&LaPO_4_: Tb^3+^, Ce^3+^ mixture was incorporated into PDMS to prepare composite elastomers, and mechanical stress was applied to these composite elastomers in a dark environment. As shown in Figure 3g, the composite elastomers emit green self‐recoverable ML under various stimuli, including scratching (I), folding (II), stretching (III), and pressing (IV). Although the ML brightness remained weak, this result can clearly demonstrate that AlF_3_ induces a contact electrification effect in the PDMS, thereby assisting in exciting the self‐recoverable ML of the LaPO_4_: Tb^3+^, Ce^3+^. Therefore, our proposed “assisting contact electrification” strategy is experimentally validated as effective. In addition, a control experiment was further conducted to rule out the possibility of AlF_3_ as the emission center. As shown in Figure S24, no self‐recoverable ML response can be observed in AlF_3_/PDMS under external mechanical stimuli. This result excludes the possibility that the detected green ML originates from AlF_3_ or the PDMS matrix itself. Figure 3h compares the PL (I) and ML (II) spectra of the AlF_3_&LaPO_4_: Tb^3+^, Ce^3+^/PDMS composite. Both spectra originate from the characteristic 4f‐4f transitions of the Tb^3+^ [81, 82, 83, 84]. The emission peaks are attributed to the ^5^D_4_ ‐^7^F_i_ (i = 6, 5, 4, 3) transitions of Tb^3+^, with the strongest peak corresponding to the green emission of ^5^D_4_ ‐^7^F_5_ [85, 86]. Notably, the ML spectrum differs distinctly from the PL spectrum due to the different measurement methods. Generally, ML is transient and faint, so its signals are collected by CCD cameras with relatively large slits, resulting in broadened emission peaks. By comparison, steady‐state photoluminescence with high luminous intensity is tested via wavelength scanning using a fluorescence spectrometer with small slits to acquire high‐precision spectral curves. Overall, the accuracy of ML spectra is inferior to that of photoluminescence spectra. In addition to variations in testing equipment, two other factors may contribute to the observed spectral differences, which were discussed in Note S2. Furthermore, Figure 3i shows the relationship between the mass content of AlF_3_ in the mixture and the self‐recoverable ML brightness of the composite. The experimental data reveal that the ML intensity of the composites increases first and then decreases with the rising addition amount of AlF_3_, and the optimal doping content is 15%. This variation trend is jointly dominated by the interfacial electrification effect, charge transport capacity, and interfacial microstructure state of the materials.

**FIGURE 4:**
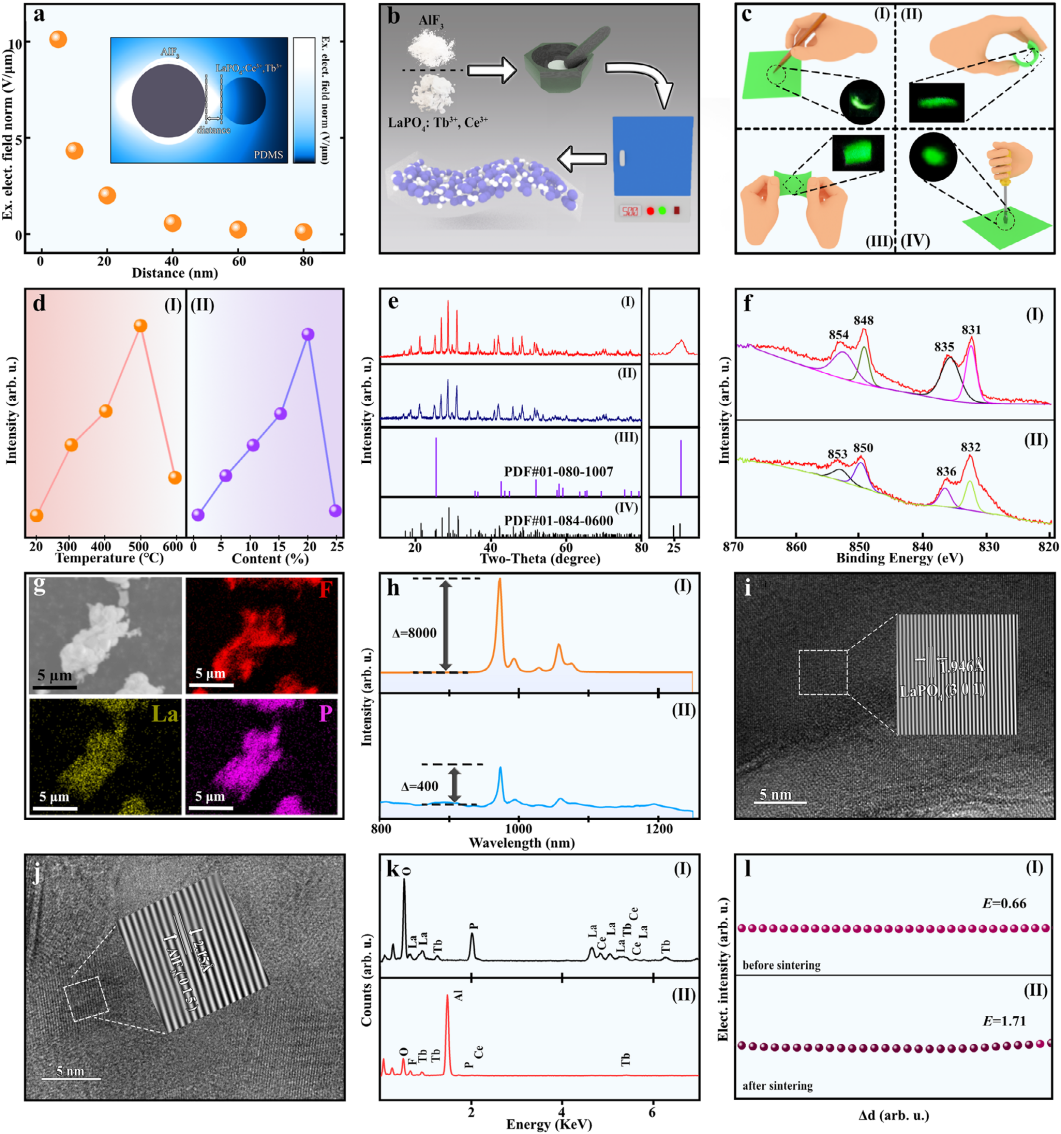
Self‐recoverable ML optimization. (a) Decay curve of the surface excitation electric field strength of the AlF_3_ and LaPO_4_ mixture in the PDMS with distance, and the finite element simulation results of the surface excitation electric field (inset); (b) Schematic diagrams: physically mixing an appropriate amount of AlF_3_ and LaPO_4_: Tb^3+^, Ce^3+^ phosphor followed by sintering treatment to prepare a composite elastomer; (c) Schematic diagrams: actions and ML photos of AlF_3_@LaPO_4_: Tb^3+^, Ce^3+^/PDMS composite elastomer under scratching (I), folding (II), stretching (III), and pressing (IV); (d) Optimization experimental results: relationships between sintering temperature (I), mass ratio of AlF_3_ (II) and the self‐recoverable ML brightness of the composite elastomer; (e) XRD patterns of samples before (I) and after (II) sintering treatment, as well as standard x‐ray diffraction lines of AlF_3_ (III) and LaPO_4_ (IV); (f) x‐ray photoelectron spectra of samples before (I) and after (II) sintering treatment; (g) Scanning electron microscope image and elemental distribution map of the sample after sintering treatment; (h) Raman spectra of samples before (I) and after (II) sintering treatment; (i) HRTEM images of the mixture of LaPO_4_: Tb^3+^, Ce^3+^ and AlF_3_ before sintering; (j) HRTEM images of the mixture of LaPO_4_: Tb^3+^, Ce^3+^ and AlF_3_ after sintering; (k) EDS line scanning profiles of the mixture of LaPO_4_: Tb^3+^,Ce^3+^ and AlF_3_ before (I) and after (II) sintering; (l) Surface excitation electric field strengths of samples before (I) and after (II) sintering treatment.

Although mixing AlF_3_ can enable the LaPO_4_: Tb^3+^, Ce^3+^, which originally has no ML of Tb^3+^, to emit self‐recoverable ML of Tb^3+^, its ML brightness remains weak. The inset of Figure 4a shows the finite element simulation results of the excitation electric field generated by physically mixed AlF_3_ and LaPO_4_ particles within the PDMS. These results show that the excitation electric field is predominantly localized at the AlF_3_/PDMS interface. In the typical physical mixtures, the AlF_3_ and LaPO_4_ particles are separated by a finite distance, and the electric field strength decays rapidly with increasing separation (Figure 4a). As a result, only a small fraction of phosphor particles in close proximity to AlF_3_ (where *E_e_* > 1 V/µm) experience sufficient field strength for effective excitation, leading to the observed low ML brightness. Therefore, an effective method must be developed to achieve closer contact between AlF_3_ and LaPO_4_: Tb^3+^, Ce^3+^ particles, minimizing the distance between the excitation source and luminescent centers, thereby enhancing the ML intensity.

Accordingly, the above mixture was sintered at appropriate temperatures to promote closer adhesion of AlF_3_ onto the surface of LaPO_4_: Tb^3+^, Ce^3+^ particles. As shown in Figure 4b, the LaPO_4_: Tb^3+^, Ce^3+^ was uniformly mixed with a certain amount of AlF_3_ (mass ratios ranging from 5% to 25%) in a mortar. The resulting mixture was then placed into a crucible and sintered in a muffle furnace at 100–800°C for 2 h. Subsequently, the obtained AlF_3_@LaPO_4_: Tb^3+^, Ce^3+^ mixture was incorporated into the PDMS to prepare a composite elastomer, and various mechanical stresses were applied to the elastomer in a dark environment. As shown in Figure 4c, the composite elastomer can emit intense green self‐recoverable ML under scratching (I), folding (II), stretching (III), and pressing (IV). Notably, this ML brightness is significantly higher than that of the unsintered sample. In addition, optimization experiments presented in Figure 4d show that the optimal sintering temperature is 500°C, and the optimal mass content of AlF3 is approximately 20%. In order to verify the influence of the sintering process itself on the phosphors, we conducted a controlled test. Pure phosphors subjected only to sintering without any assisting electrification additives were compounded with the PDMS matrix. As displayed in Figure S25, no ML was observed in the obtained composites. Meanwhile, the KPFM spectra in Figure S26 shows that the two phosphor samples before and after sintering exhibit negligible surface potential difference and nearly identical potential levels. The results fully verify that sintering treatment at 500°C cannot effectively enhance the contact electrification capability of the phosphors. Moreover, we have supplemented mechanical tests on pure PDMS elastomer and LaPO_4_: Tb^3+^,Ce^3+^@AlF_3_/PDMS composites, including tensile strength, elongation at break, elastic modulus, and cyclic tensile fatigue property. The results were exhibited in Note S3. The test results reveal that the introduction of LaPO_4_:Tb^3+^,Ce^3+^ and AlF_3_ can moderately enhance the mechanical strength and rigidity of the PDMS matrix, without obvious deterioration in ductility.

Figure 4e and Figures S27–S31 show the XRD patterns of the AlF_3_&LaPO_4_: Tb^3+^, Ce^3+^ samples before and after sintering treatment. It indicates that after sintering at 500°C for 2 h, the characteristic XRD diffraction peaks of AlF_3_ can still be clearly observed without significant changes in intensity. This result indicates that the sintering treatment does not significantly change the phase composition of the mixture samples. Meanwhile, XPS results in Figure 4f indicate that the sintering treatment does not significantly change the ion valence states of Tb^3+^ and Ce^3+^ on the surface of the LaPO_4_: Tb^3+^, Ce^3+^. Figure 4g shows the SEM images and elemental mapping of the sintered samples. It can be seen that most of the AlF_3_ is relatively uniformly distributed on the surface of the LaPO_4_: Tb^3+^, Ce^3+^ particles. Does this imply closer interfacial contact between AlF_3_ and the phosphor particles after sintering? According to the Raman spectra in Figure 4h, the intensity of the characteristic Raman peaks of LaPO_4_ is significantly reduced after sintering. This reduction arises from partial diffusion of AlF_3_ into the surface lattice of the LaPO_4_ during high‐temperature treatment, forming a trace solid solution. Although this minor incorporation does not affect the dominant phases of AlF_3_ and LaPO_4_ in the mixture, it disrupts the surface lattice periodicity of the LaPO_4_, thereby reducing the Raman scattering efficiency of the LaPO_4_. This observation provides direct evidence that the interfacial contact between the AlF_3_ and LaPO_4_: Tb^3+^, Ce^3+^ becomes significantly tighter after sintering. Consequently, the distance between the excitation source and the luminescent centers is shortened in the composite, leading to enhanced ML intensity. As shown in HRTEM images of Figure 4i, before sintering, clear and continuous lattice fringes assigned to the (301) crystal plane of LaPO_4_ can be distinctly observed on the surface of LaPO_4_: Tb^3+^, Ce^3+^ particles in the physically mixed sample, and no obvious AlF_3_ phase is detected. In contrast, after sintering, characteristic lattice fringes corresponding to the (015) plane of AlF_3_ appear on the LaPO_4_: Tb^3+^, Ce^3+^ particle surface in Figure 4j.

Meanwhile, EDS line scanning characterization was carried out. As presented in Figure 4k‐I, only intrinsic elemental signals of LaPO_4_: Tb^3+^,Ce^3+^ are detected on the particle surface before sintering, with no Al and F signals observed. After sintering, strong characteristic signals of Al and F are detected on the particle surface in Figure 4k‐II, while the signals of La, P, Tb and Ce are much weaker. These results verify that obvious interfacial diffusion of AlF_3_ occurs during high‐temperature sintering, and AlF_3_ uniformly coats the surface of LaPO_4_: Tb^3+^,Ce^3+^ particles. Additionally, Figure 4l shows that the surface excitation electric field strength of the mixture after sintering increases significantly from an initial 0.66–1.71, corresponding to the enhancement of contact electrification. Collectively, these findings confirm the effectiveness of our proposed “assisting contact electrification strategy”, although further validation across additional phosphors is still required.

Furthermore, it suggests that future research can focus on inactive commercial phosphors with high luminous efficiency, stable physicochemical properties, and weak contact electrification capability. As for the assisting electrification materials, fluorides such as CaF_2_, MgF_2_, SrF_2_, and oxides including SiO_2_, Al_2_O_3_, MgO, and WO_3_ all feature high relative surface work function, which may follow the same working mechanism as AlF_3_. As for the assisting method, we consider that the wet‐chemical coating methods (such as co‐precipitation, sol‐gel, and evaporative crystallization) are highly feasible for constructing ultra‐thin, uniform, and dense active assisting electrification material shells on phosphor surfaces. Such structures can effectively shorten charge transfer paths, reduce interfacial charge loss, and ultimately achieve superior self‐recoverable ML performance. We believe that the assisting electrification strategy proposed in this work can be theoretically extended to address the scarcity of piezoelectric‐induced ML materials. Specifically, functional materials with high piezoelectric activity can be introduced as assisting electrification and energy‐supplying units to modify phosphors with weak or negligible piezoelectric response via composite construction and interface regulation. Under mechanical stress, the highly piezoelectric components generate charges and built‐in electric fields, while the phosphors serve as efficient luminescent centers to realize light emission. In this way, the synergistic coupling mechanism of “piezoelectric energy supply → interfacial charge transfer → energy‐level emission” is achieved. At this stage, we temporarily refer to the above interesting idea as “**Zhang's Conjecture**.” We hope it will inspire fellow researchers and eventually solve the scarcity problem of piezoelectric‐induced ML materials.

## 3 Conclusions

LaPO_4_: Tb^3+^, Ce^3+^, which possesses a centrosymmetric crystal structure, does not exhibit piezoelectrically excited ML. Significantly, due to its weak contact electrification capability, LaPO_4_: Tb^3+^, Ce^3+^ cannot be effectively excited to emit ML of Tb^3+^ through the contact electrification effect in the PDMS. To address this limitation, an appropriate amount of strongly electrifiable AlF_3_ was mixed in the composite as an assisting material. Assisted by AlF_3_‐induced strong contact electrification, the self‐recoverable ML of Tb^3+^ for the LaPO_4_: Tb^3+^, Ce^3+^ was successfully excited. Furthermore, sintering treatment promotes partial diffusion of the interfacial AlF_3_ into the surface lattice of the LaPO_4_: Tb^3+^, Ce^3+^, shortening the distance between the excitation source and luminescent center, thereby further improving its self‐recoverable ML brightness. This case indicates that the originally rare self‐recoverable ML may become a universal property of all phosphors. It provides an important new strategy for the development of novel high‐efficiency self‐recoverable ML materials.

## 4 Experimental Section

### 4.1 Mixing Treatment of LaPO_4_: Tb^3+^, Ce^3+^ Phosphor

The LaPO_4_: Tb^3+^, Ce^3+^ phosphor used in this experiment was purchased from Gansu Rare Earth New Materials Co., Ltd. The mixing treatment process is as follows:

1. Raw particles: LaPO_4_: Tb^3+^, Ce^3+^ was filtered through a 100–200 mesh sieve to remove agglomerates.
2. Physical mixing: The resulting LaPO_4_: Tb^3+^, Ce^3+^ was fully mixed with AlF_3_ particles in an agate mortar at a certain ratio.
3. Mixed sintering: The resulting mixture of LaPO_4_: Tb^3+^, Ce^3+^ and AlF_3_ particles was placed in a crucible, calcined at 100–1000°C for 2 h, and naturally cooled to room temperature.

### 4.2 Preparation of Phosphor/PDMS Composite

Sylgard 184 silicone rubber produced by Dow Corning was selected as the matrix material. The specific preparation process is as follows: Mix liquid A and liquid B at a certain ratio (liquid B accounts for 5%–20% by mass); add a certain amount of phosphor to the obtained mixed slurry (phosphor accounts for 30%–50% by mass) and stir thoroughly to ensure uniform dispersion of the phosphor; finally, transfer the resulting mixed slurry to a Petri dish and cure it in a constant temperature oven at 60–80°C for 2–6 h to obtain the desired ML composite material.

### 4.3 Characterizations

X‐ray diffraction (XRD) patterns of the samples were measured using an x‐ray diffractometer (Rigaku D/Max‐2400). The morphology (SEM) of sample particles and energy dispersive spectroscopy (EDS) from selected spots were recorded using a scanning electron microscope (FEI ApreoS). The photoluminescence (PL) spectra of the samples were measured using a fluorescence spectrometer (Omni‐λ300i) with a 500 W xenon lamp as the excitation source. The Raman spectra of the samples were characterized using a Raman spectrometer (HORIBA; LabRAM Odyssey). The surface morphology (AFM) of the samples was obtained using an atomic force microscope (Bruker; ICON). The rare earth ions were analyzed using an x‐ray photoelectron spectrometer (PHI‐5702). ML signals were collected in situ through a collimator connected to an optical fiber (BFC‐441; Zolix Instruments Co., Ltd.) and detected using a CCD camera (iVac‐316; Edmund Optics Ltd.) and a spectrometer (Omni‐A300i; Zolix Instruments Co., Ltd.). All photos and videos were taken with a Huawei P40 Pro mobile phone, and all characterization tests were performed at room temperature.

## Author Contributions

**Weiguang Wang**: methodology, data curation, validation, investigation, visualization, software. **Jianwen Zhang**: conceptualization, validation, methodology, data curation, supervision, writing – original draft, visualization, software, formal analysis, investigation, writing – review and editing. **Haoyang Li**: software, data curation, methodology, validation, investigation, visualization. **Wenxiang Wang**: conceptualization, methodology, software, data curation, supervision, formal analysis, validation, investigation, visualization, writing – review and editing. **Shanwen Wang**: investigation, methodology, validation, data curation, visualization. **Jiachi Zhang**: project administration, resources, writing – review and editing, writing – original draft, funding acquisition, conceptualization, methodology. Chi Zhang: investigation, visualization, software, validation. **Zhaofeng Wang**: funding acquisition, project administration, resources. **Xianfeng Jin**: investigation, software, visualization. **Yuhua Wang**: funding acquisition, project administration, resources. **Jinyu Zhou**: software, methodology, investigation, visualization, validation, data curation. **Jing Liu**: investigation, methodology, validation, data curation, software. **Ziyuan Li**: investigation, visualization.

## Funding

The National Natural Science Foundation of China (No. 10904057, 12074159 and 52272154), the Science and Technology Projects of Gansu Province (No. 18JR3RA270), the Hui‐Chun Chin and Tsung‐Dao Lee Chinese Undergraduate Research Endowment (No. JZH2825) the Taishan Scholars Program, the Key Program of the Natural Science Foundation of Gansu Province (25JRRA471), and The Fundamental Research Funds for the Central Universities (lzujbky‐2024‐25).

## Conflicts of Interest

The authors declare no conflicts of interest.

## Supporting information




**Supporting File**: advs76254‐sup‐0001‐SuppMat.docx.

## Data Availability

The data that support the findings of this study are available from the corresponding author upon reasonable request.
