# Supplementary material for: An Assisting Contact Electrification Strategy for Achieving Self‐Recoverable Mechanoluminescence
Source: Adv Sci (Weinh). 2026 Jun 30:e76254. Online ahead of print. doi: 10.1002/advs.76254 (PMC13336422; doi:10.1002/advs.76254)
Supplement: Supplementary file 1 — Supporting File: advs76254‐sup‐0001‐SuppMat.docx. [file ADVS-9999-e76254-s001.docx]

Supporting Information

**An assisting contact electrification strategy for achieving self-recoverable mechanoluminescence**

Jianwen Zhang^1#^, Weiguang Wang^1#^, Jinyu Zhou^1, 3, 4#^, Jiachi Zhang^1^*, Wenxiang Wang^1, 2^*, Shanwen Wang^1^, Jing Liu^1^, Haoyang Li^1^, Xianfeng Jin^1, 3, 4^,

Chi Zhang^1, 3, 4^, Ziyuan Li^1, 3, 4^, Zhaofeng Wang^3, 4^*, Yuhua Wang^1^*

1. National & Local Joint Engineering Laboratory for Optical Conversion Materials and Technology, Lanzhou University, Lanzhou 730000, P.R. China

2. Key Laboratory of Anisotropy and Texture of Materials (Ministry of Education), School of Materials Science and Engineering, Northeastern University, Shenyang 110819, P.R. China

3. Shandong Laboratory of Advanced Materials and Green Manufacturing at Yantai, Yantai 264006, P.R. China

4. State Key Laboratory of Solid Lubrication, Lanzhou Institute of Chemical Physics, Chinese Academy of Sciences, Lanzhou 730000, P.R. China

# These authors contribute equally to this work.

*Corresponding author. Jiachi Zhang Email: zhangjch@lzu.edu.cn

Wenxiang Wang Email: wangwx@mail.neu.edu.cn

Zhaofeng Wang Email: zhfwang@licp.cas.cn

Yuhua Wang Email: wyh@lzu.edu.cn

**This PDF file includes:**

Figs. S1 to S34

Tables S1 to S5

**Supplementary Note1 to Note3**


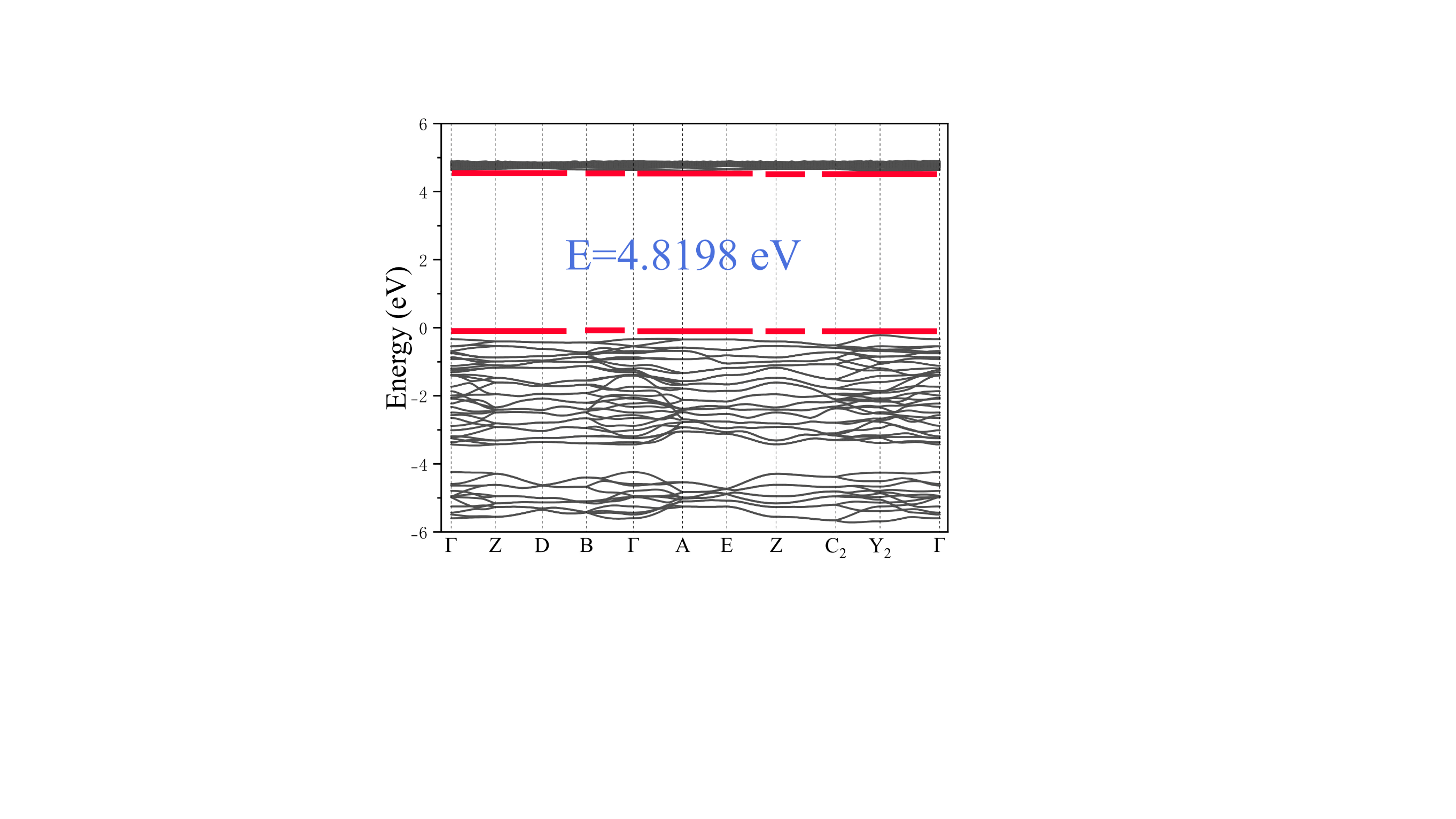


Fig. S1. Electronic structure of the LaPO_4_.

The electronic structure of LaPO_4_ indicates that LaPO_4_ has an indirect band gap, and its band gap is as wide as 4.82 eV. This result suggests that LaPO_4_ has low electrical conductivity and is difficult to form a stable piezoelectric response.


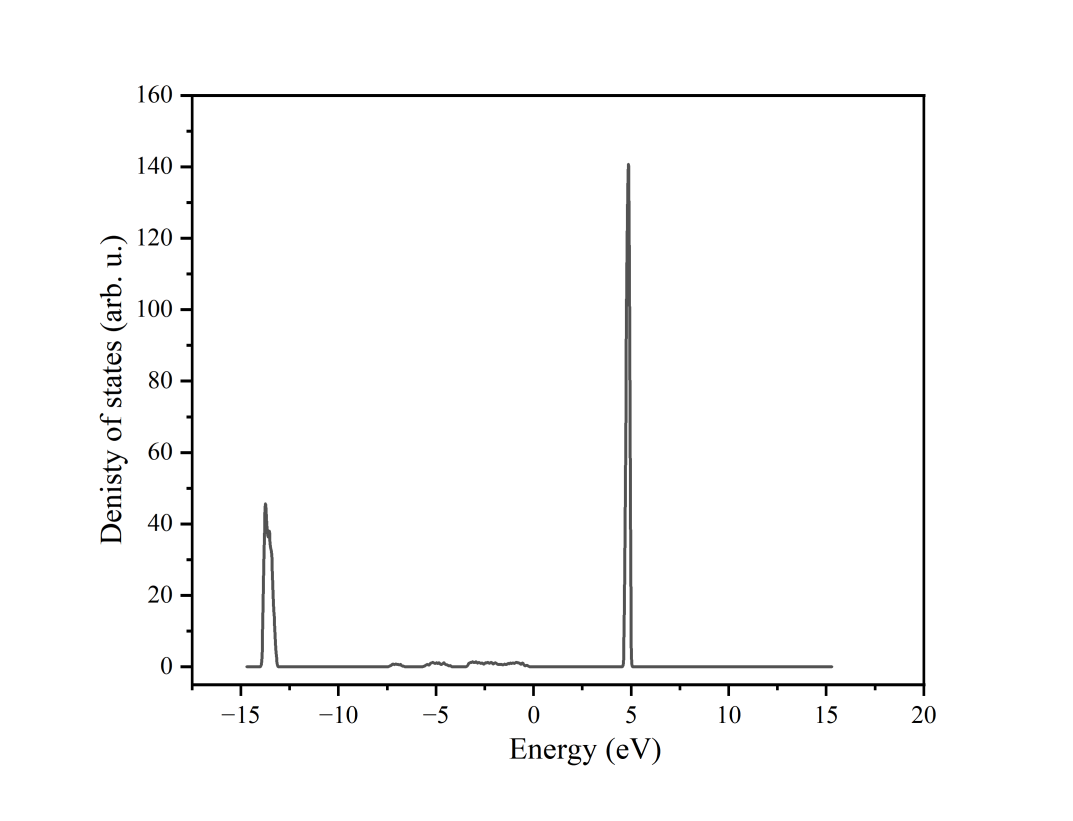


Fig. S2. DOS of La element.


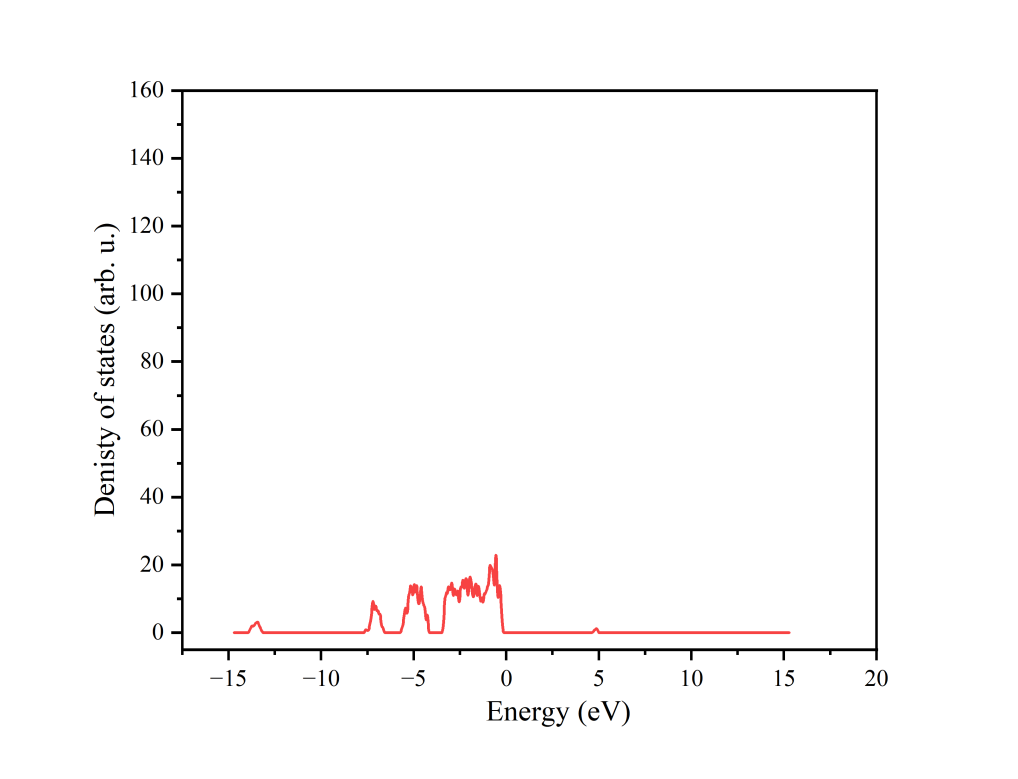


Fig. S3. DOS of O element.


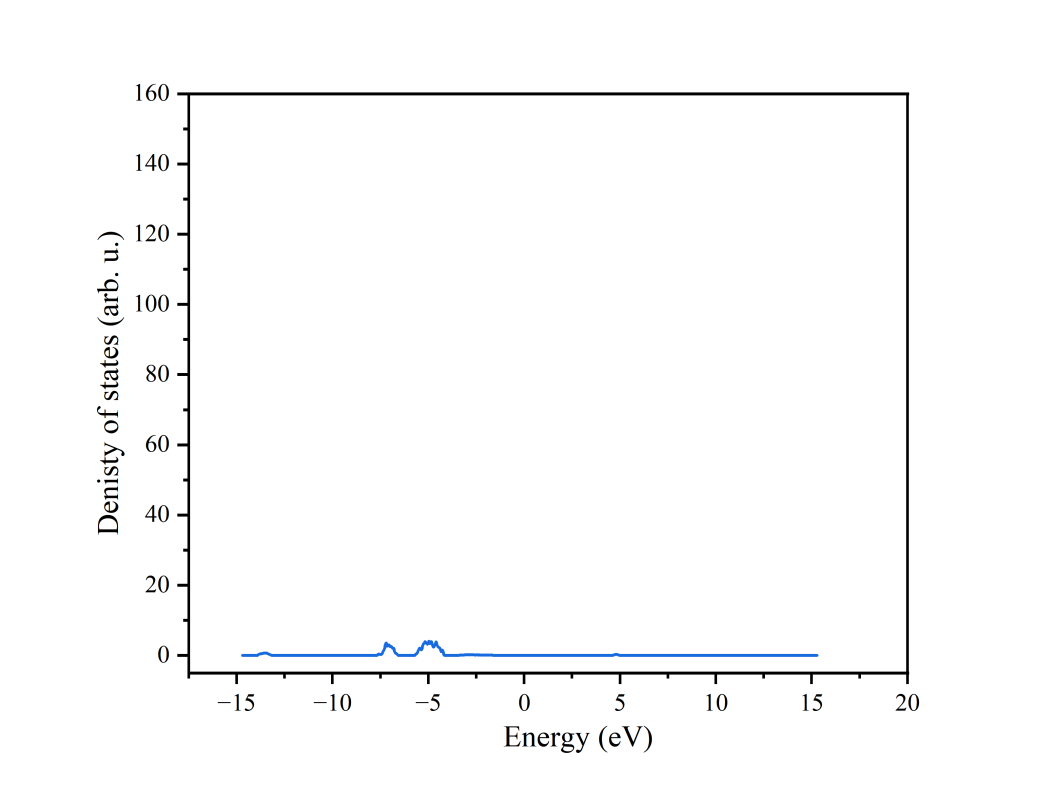


Fig. S4. DOS of P element.


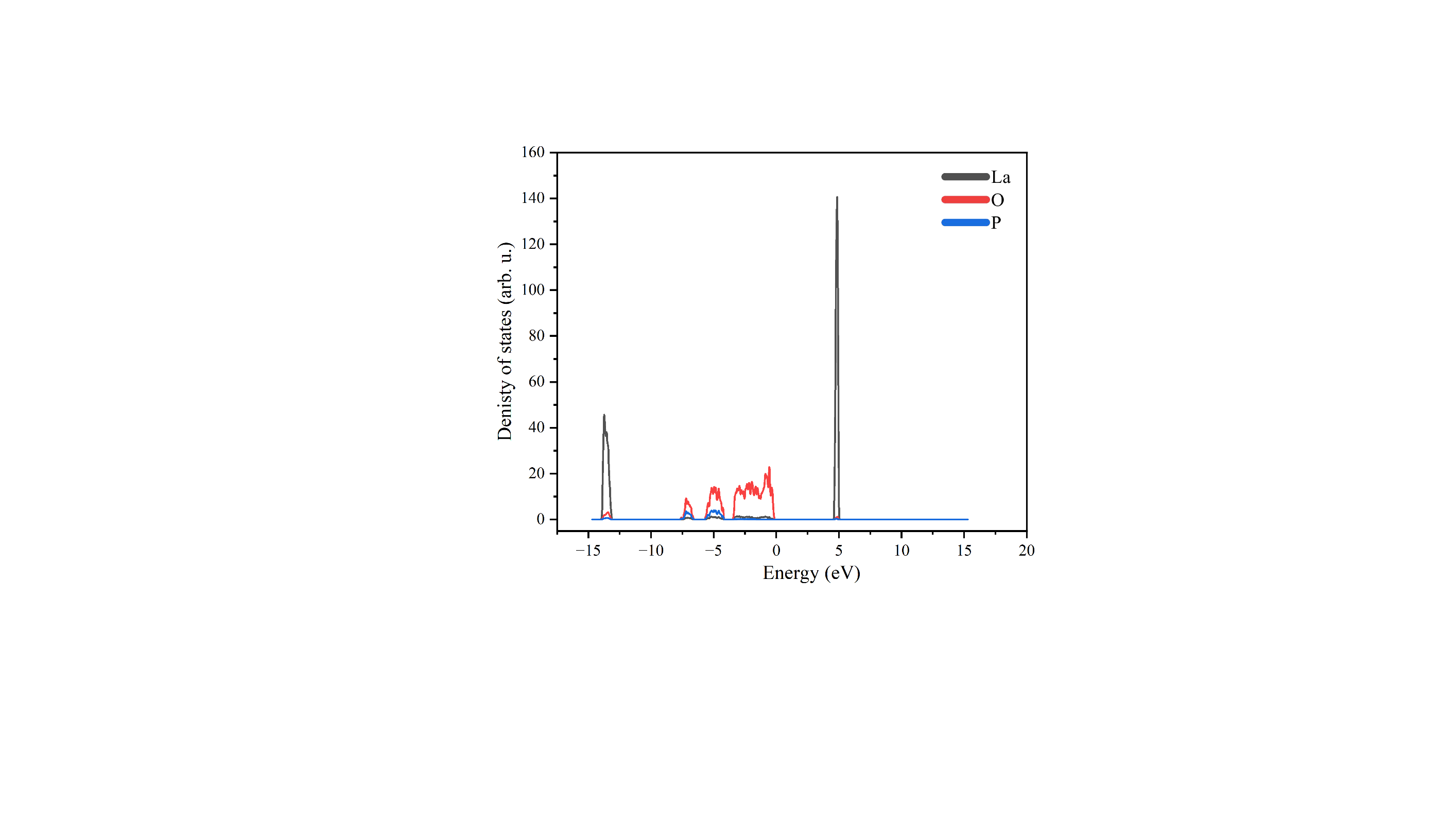


Fig. S5. DOS of LaPO_4_.


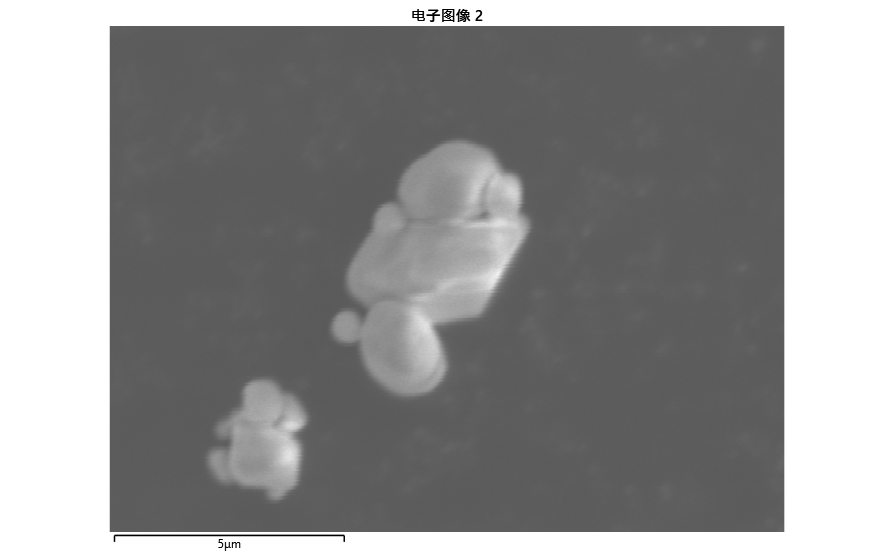


Fig. S6. SEM image of commercial LaPO_4_: Tb^3+^, Ce^3+^


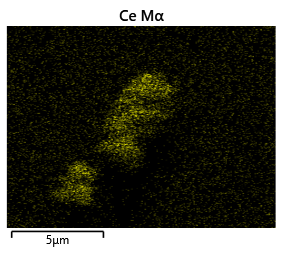


Fig. S7. Distribution of Ce element in LaPO_4_: Tb^3+^, Ce^3+^


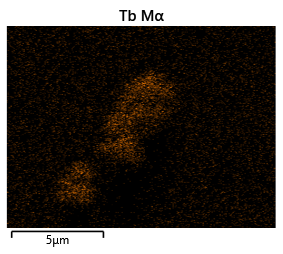


Fig. S8. Distribution of Tb element in LaPO_4_: Tb^3+^, Ce^3+^


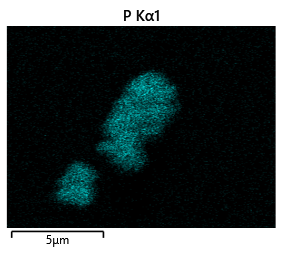


Fig. S9. Distribution of P element in LaPO_4_: Tb^3+^, Ce^3+^


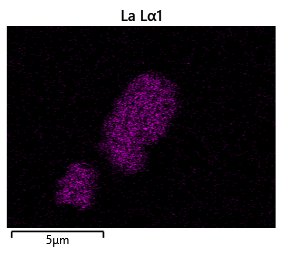


Fig. S10. Distribution of La element in LaPO_4_: Tb^3+^, Ce^3+^


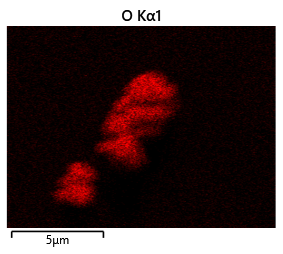


Fig. S11. Distribution of O element in LaPO_4_: Tb^3+^, Ce^3+^


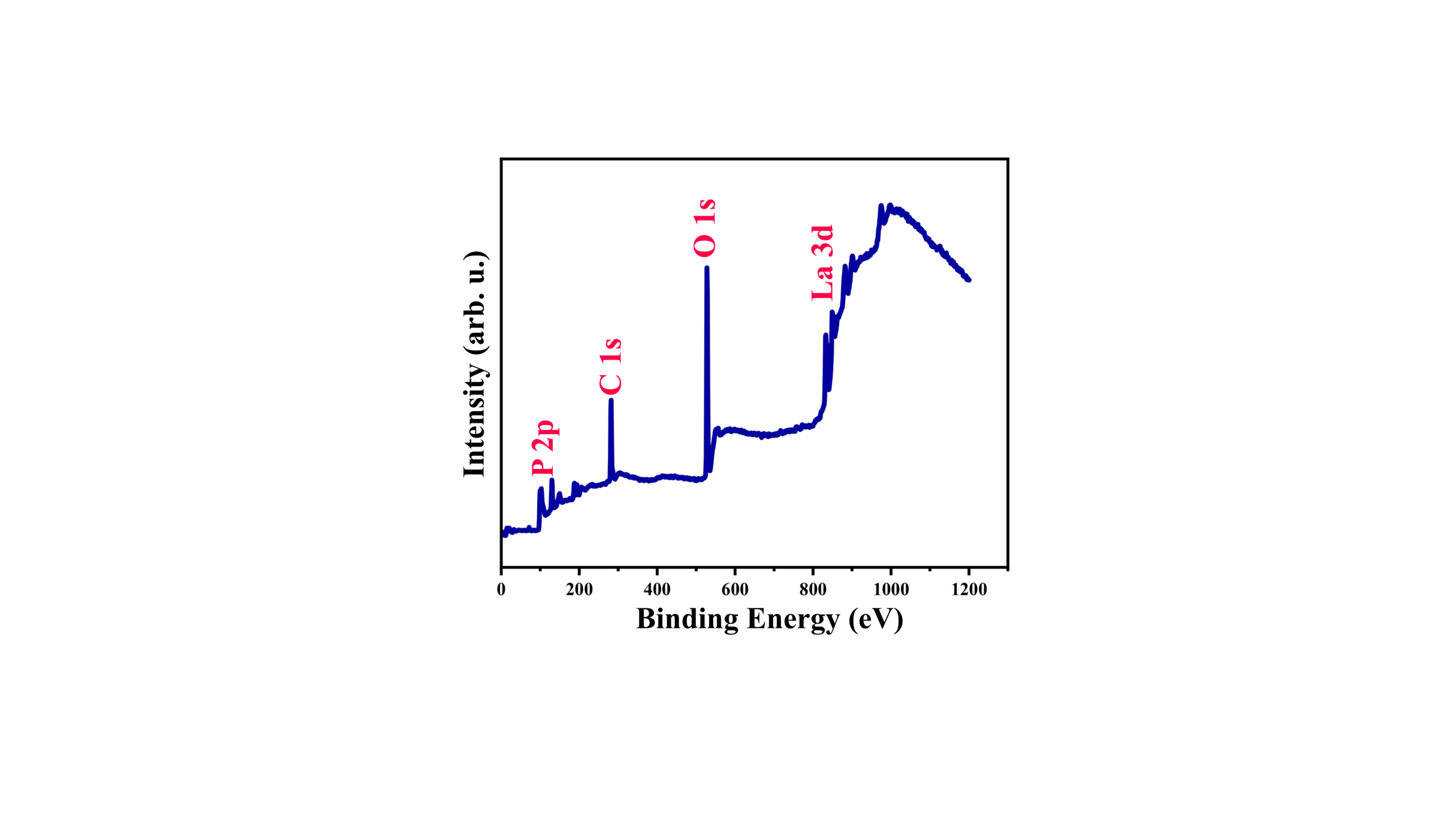


Fig. S12. XPS spectrum of LaPO_4_: Tb^3+^, Ce^3+^


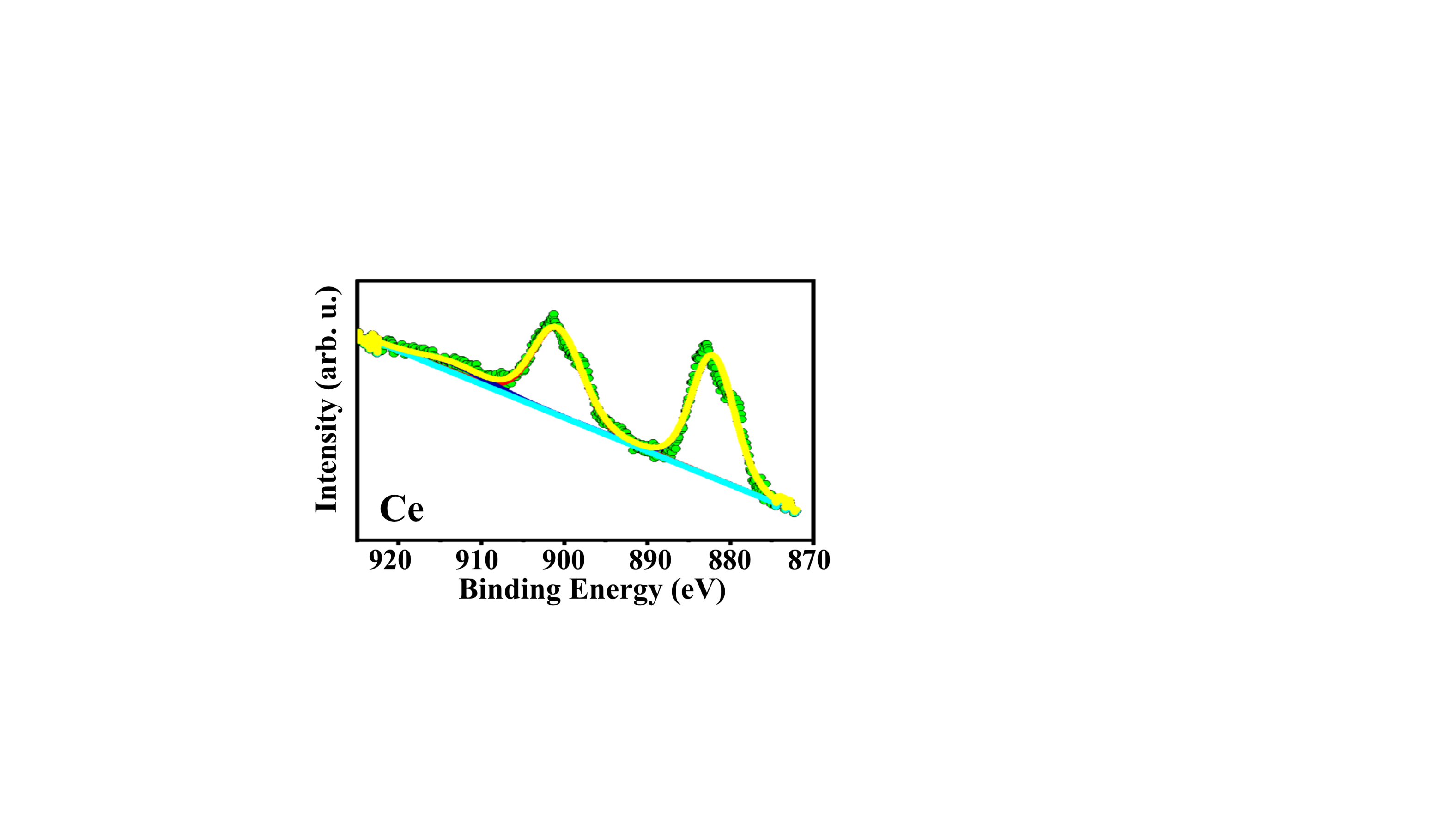


Fig. S13. XPS spectrum of Ce element in LaPO_4_: Tb^3+^, Ce^3+^


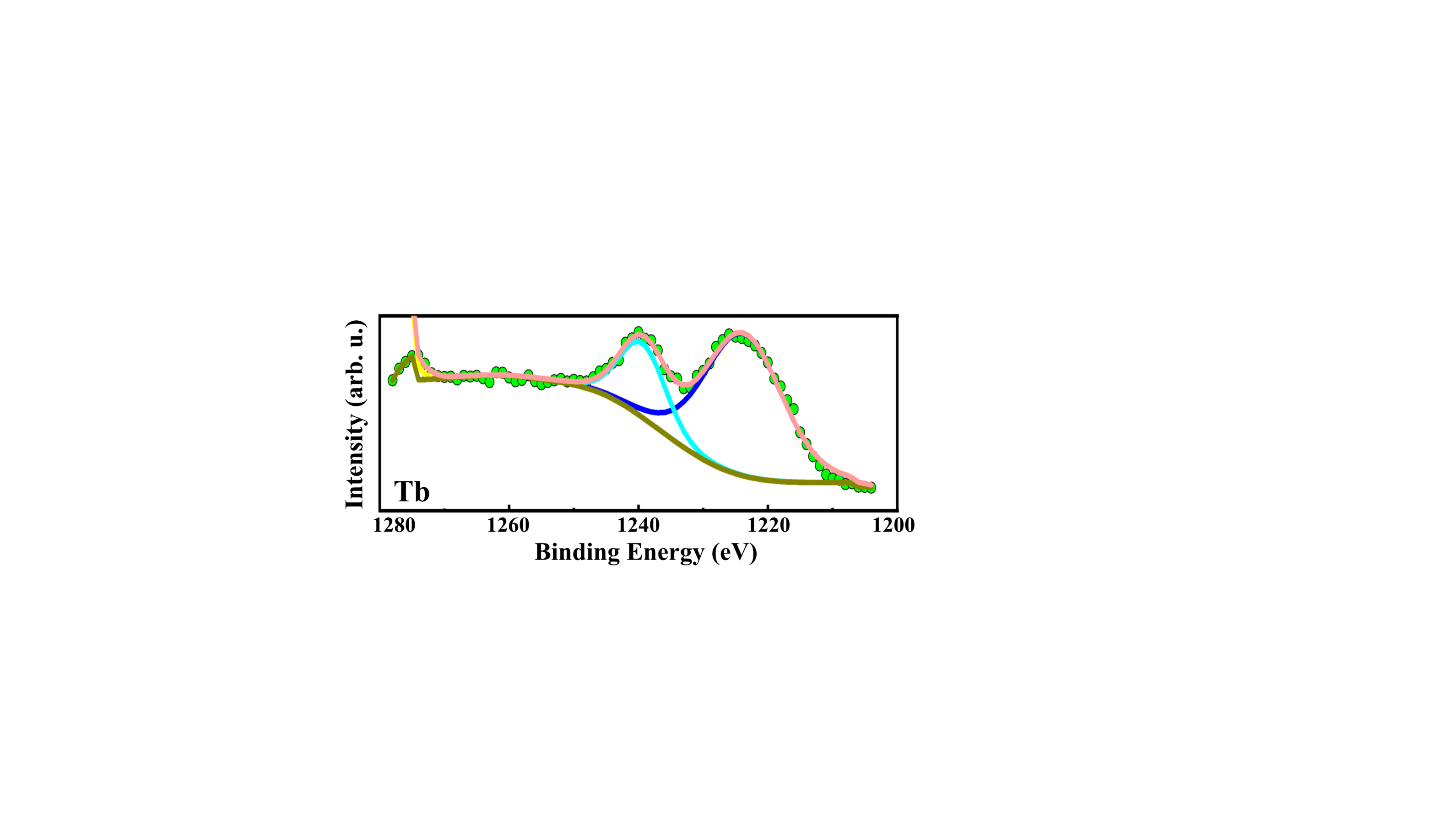


Fig. S14. XPS spectrum of Tb element in LaPO_4_: Tb^3+^, Ce^3+^

**Methods for calculating differential charge density**：

First, construct a slab model of the LaPO_4_ matrix (1×1) crystal plane, featuring a 1×1 configuration in the transverse direction and a specified number of atomic layers in the longitudinal direction. Next, develop a molecular model of the PDMS monomer, followed by a composite model combining the slab and PDMS monomer. Structural optimization is performed on each model, after which their charge densities are calculated.

Δ𝜌 = 𝜌all − 𝜌PDMS − 𝜌slab

Finally, the differential charge density is obtained.


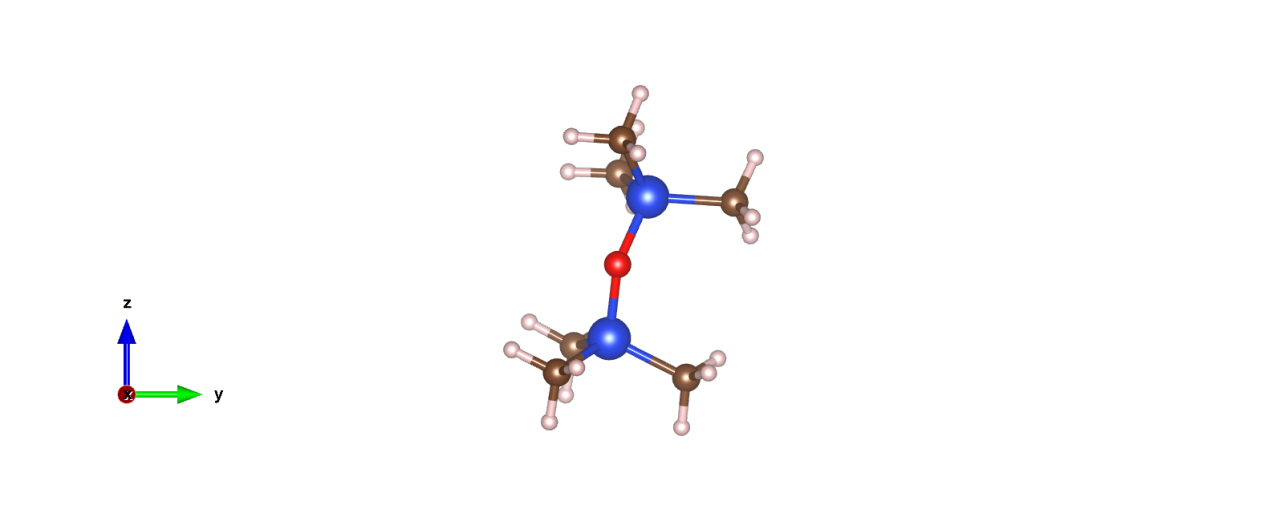


Fig. S15. Molecular model of X-view PDMS monomer


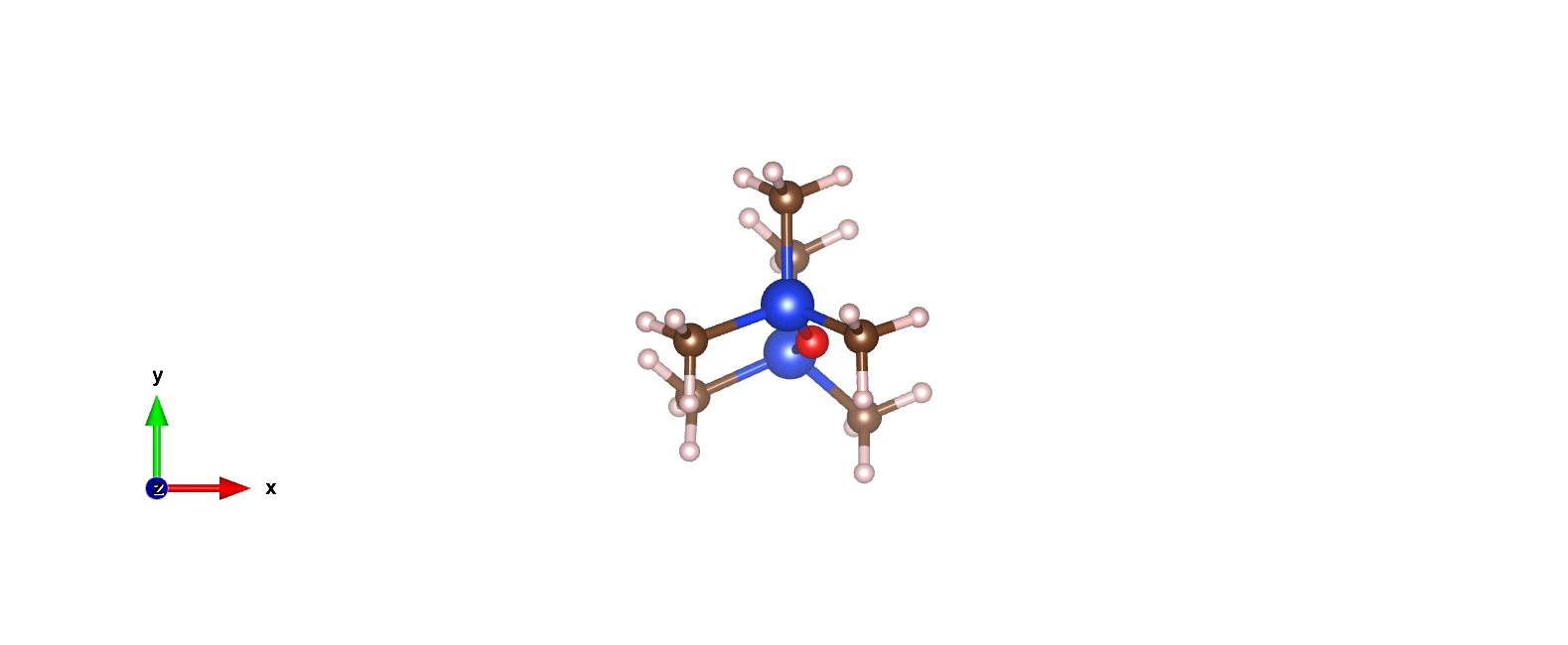


Fig. S16. Molecular model of Z-view PDMS monomer


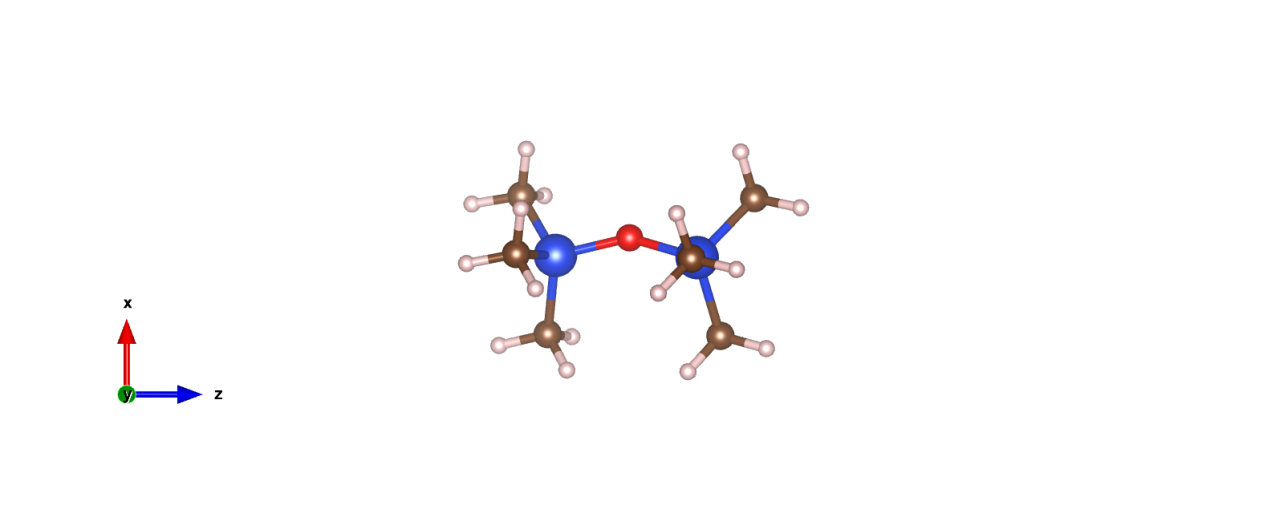


Fig. S17. Molecular model of Y-view PDMS monomer


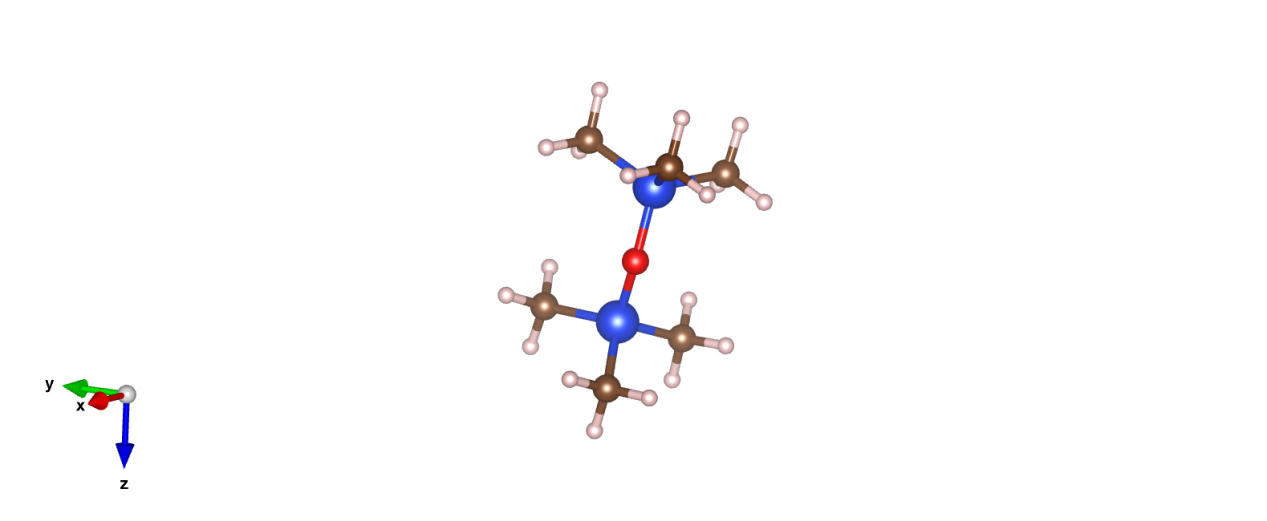


Fig. S18. Molecular model of PDMS monomer with free viewpoint


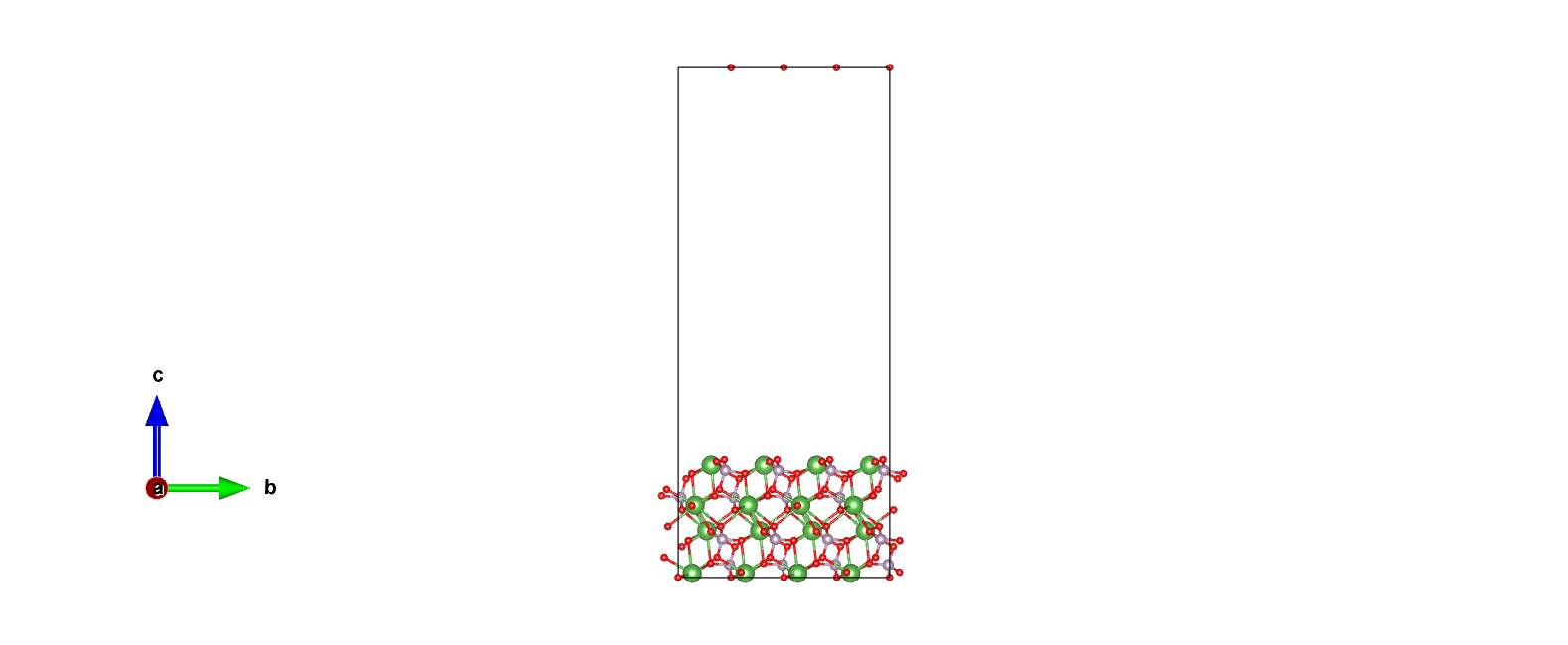


Fig. S19. Representative slab of LaPO_4_, (100) surfaces


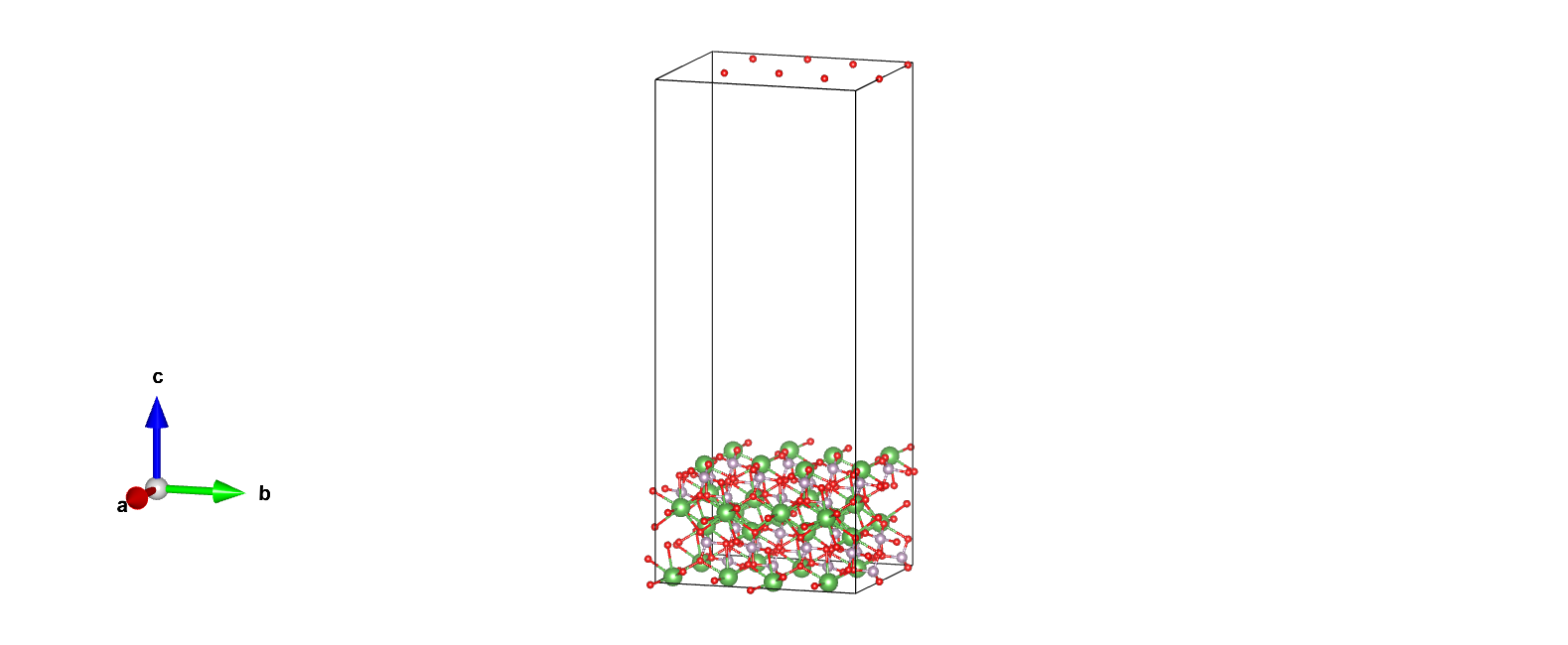


Fig. S20. Representative slab of LaPO_4_, (100) surfaces


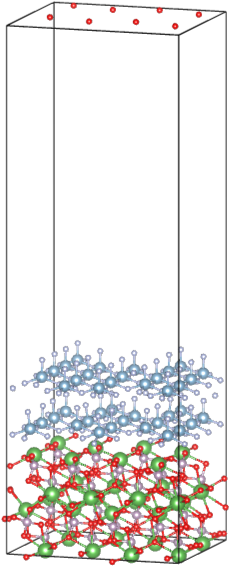


Fig. S21. Representative slab of LaPO_4_@AlF_3_


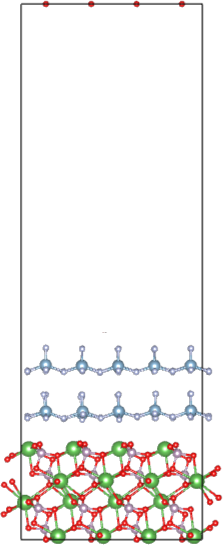


Fig. S22. Representative slab of LaPO_4_@AlF_3_


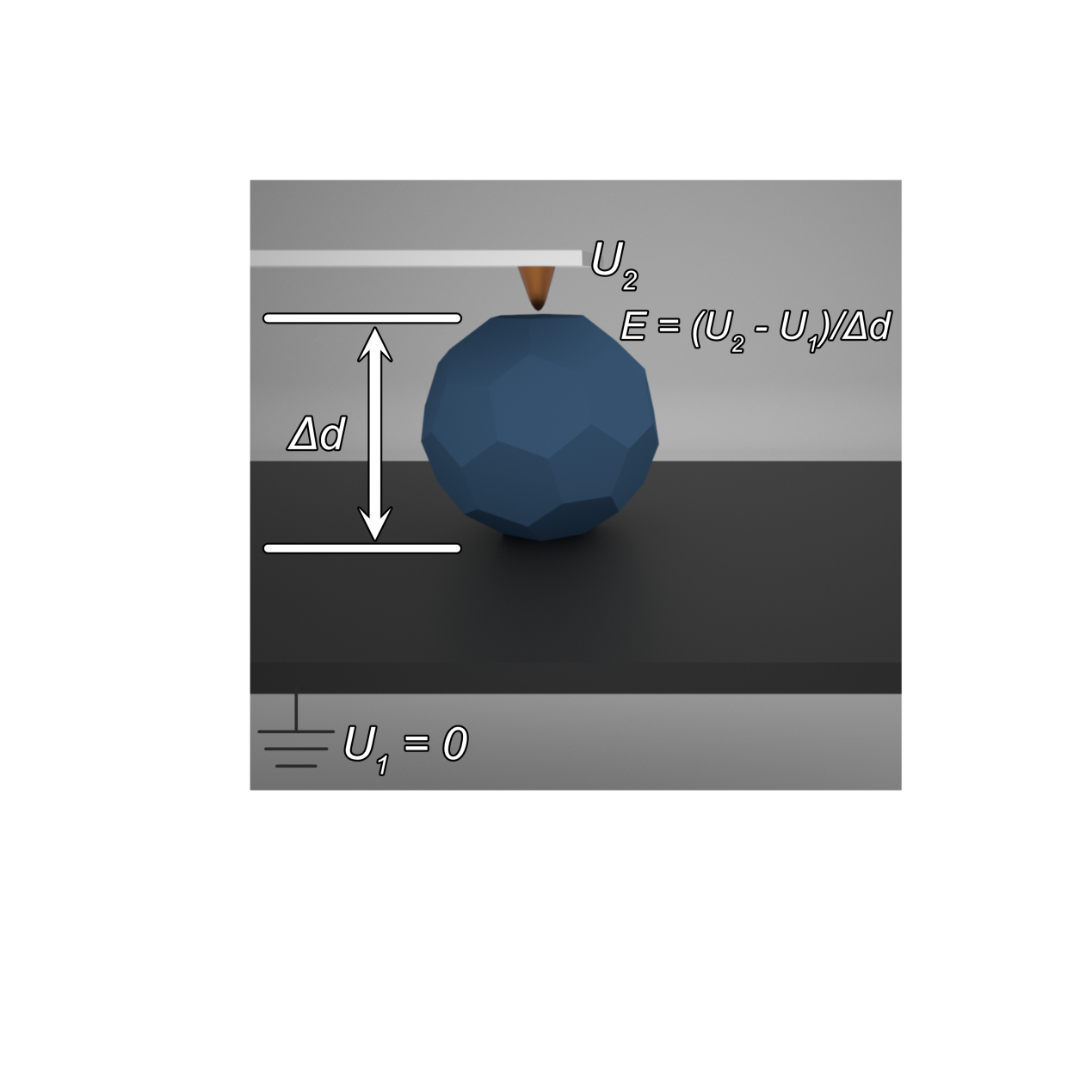


Fig. S23. Measurement and calculation methods of induced electric field intensity.


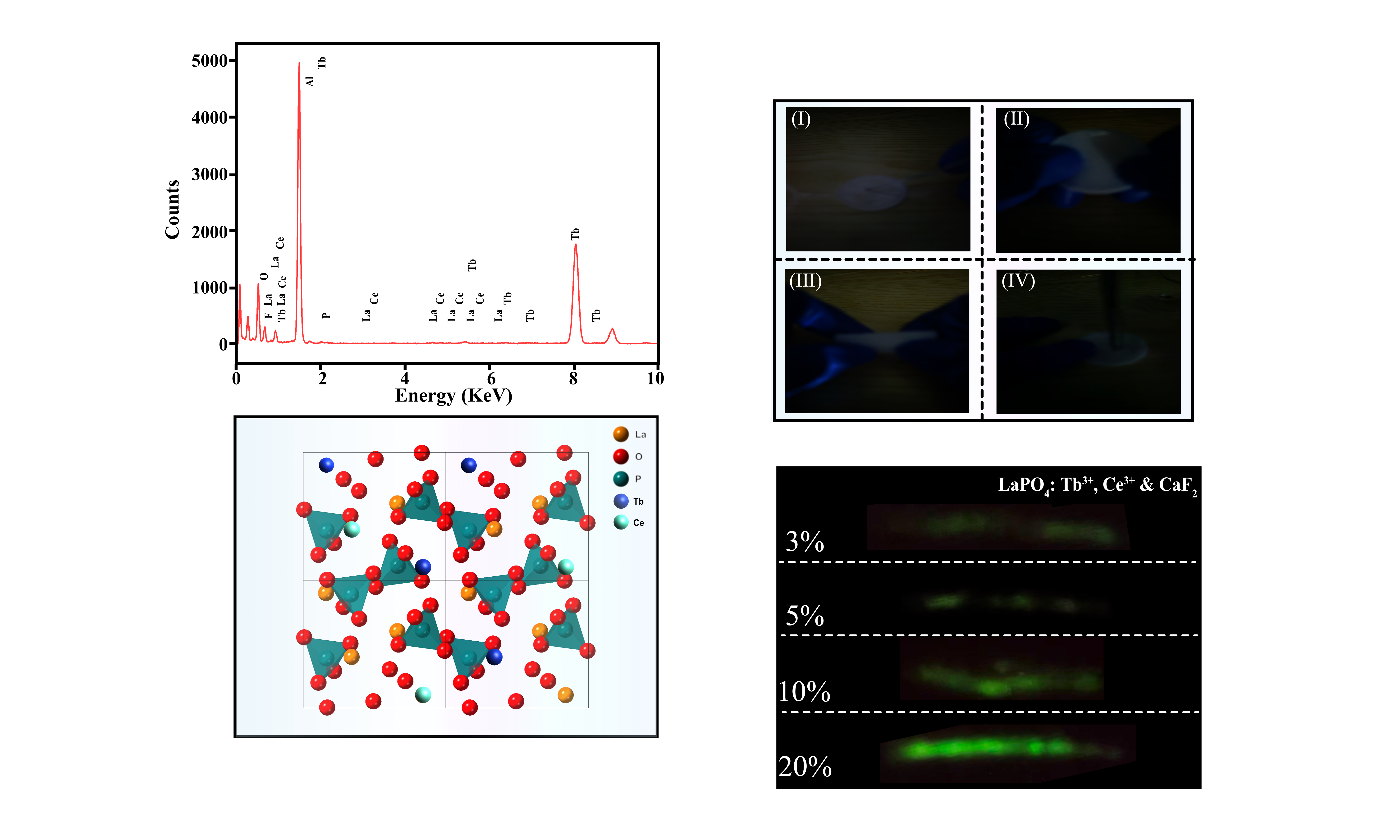


**Fig. S24.** Photographs of AlF_3_/PDMS composite under scratching (I), stretching (II), folding (III) and pressing (IV) stimuli


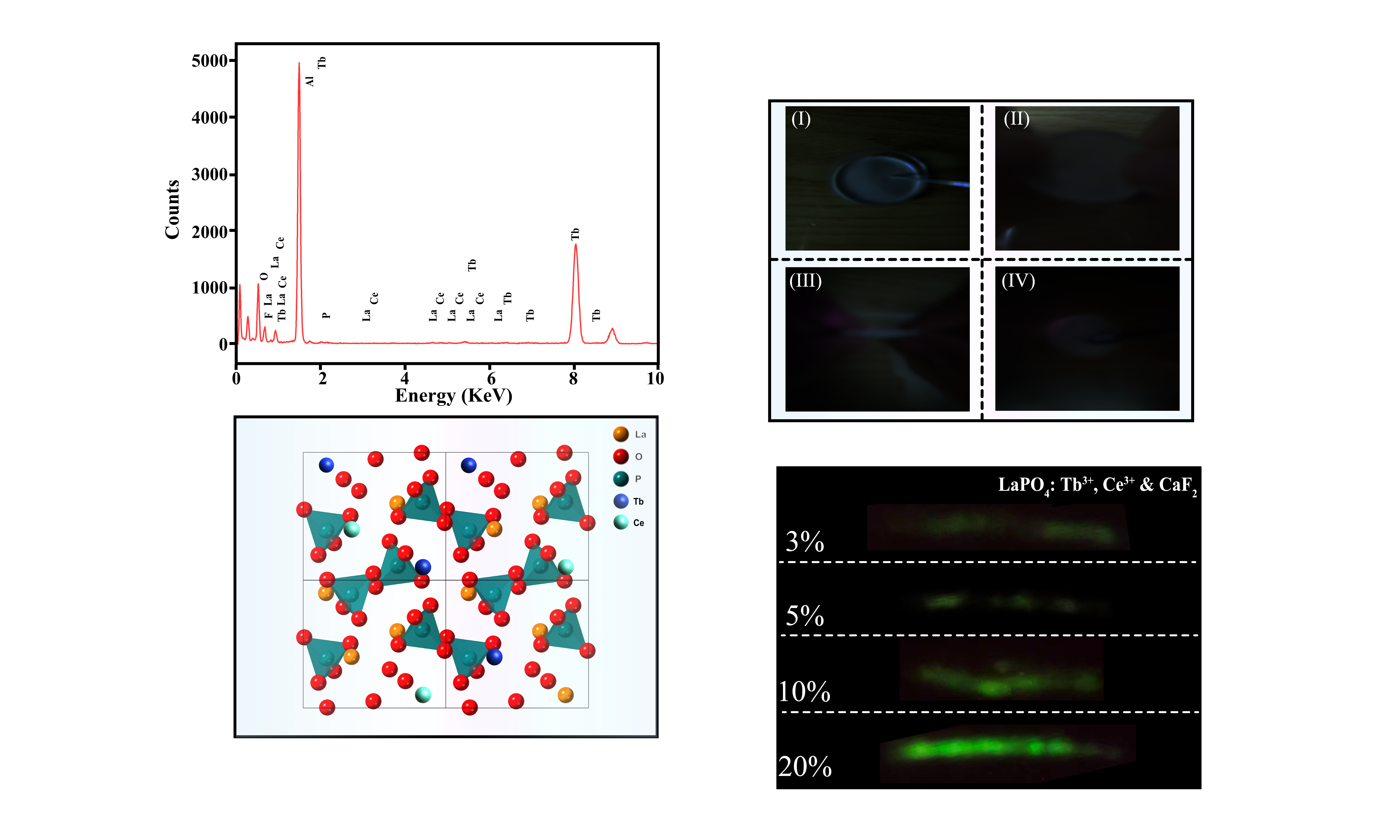


**Fig S25** Photographs of composites containing 500 °C-sintered pure LaPO_4_:Tb^3+^,Ce^3+^ phosphor under different mechanical stimuli: (I) scratching, (II) stretching, (III) folding and (IV) pressing (no ML is observed).


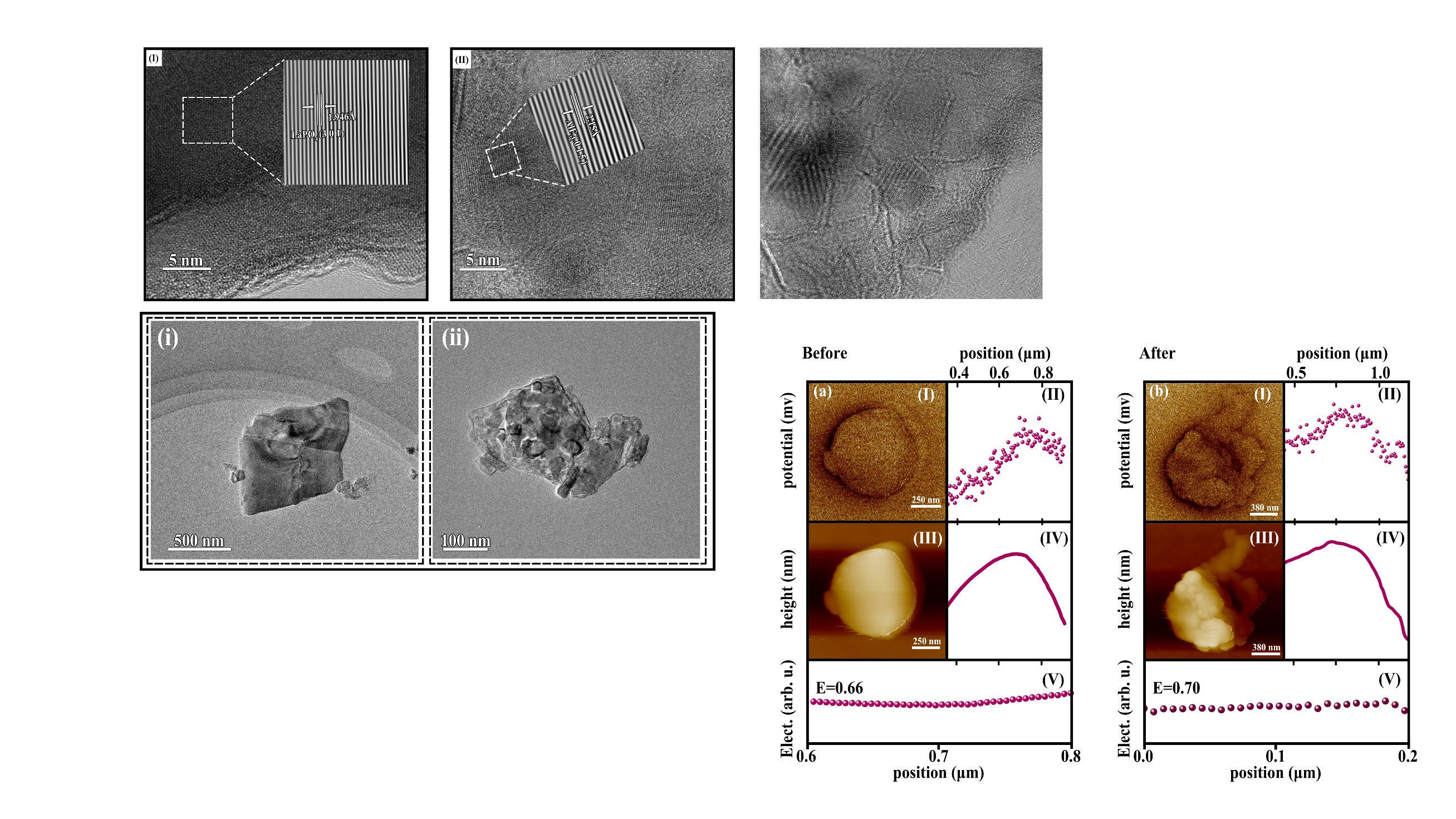


**Fig S26.** KPFM potential distribution (I), potential profile (II), AFM morphology (III), height distribution (IV) and electric field intensity distribution (V) of LaPO_4_: Tb^3+^, Ce^3+^ before and after sintering treatment.


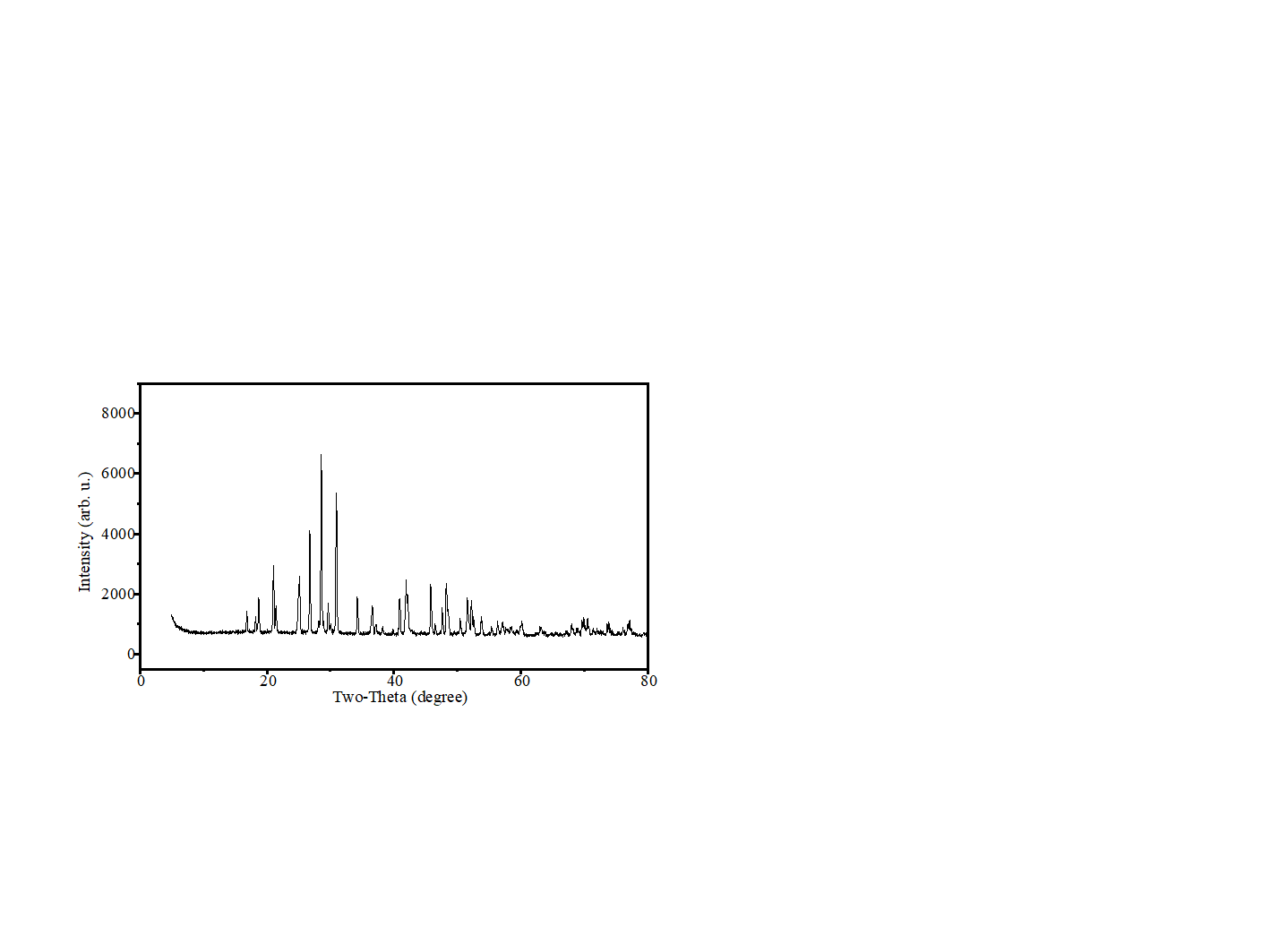


Fig. S27. XRD spectra of LaPO_4_: Tb^3+^, Ce^3+^& AlF_3_ samples sintered at 200 ℃


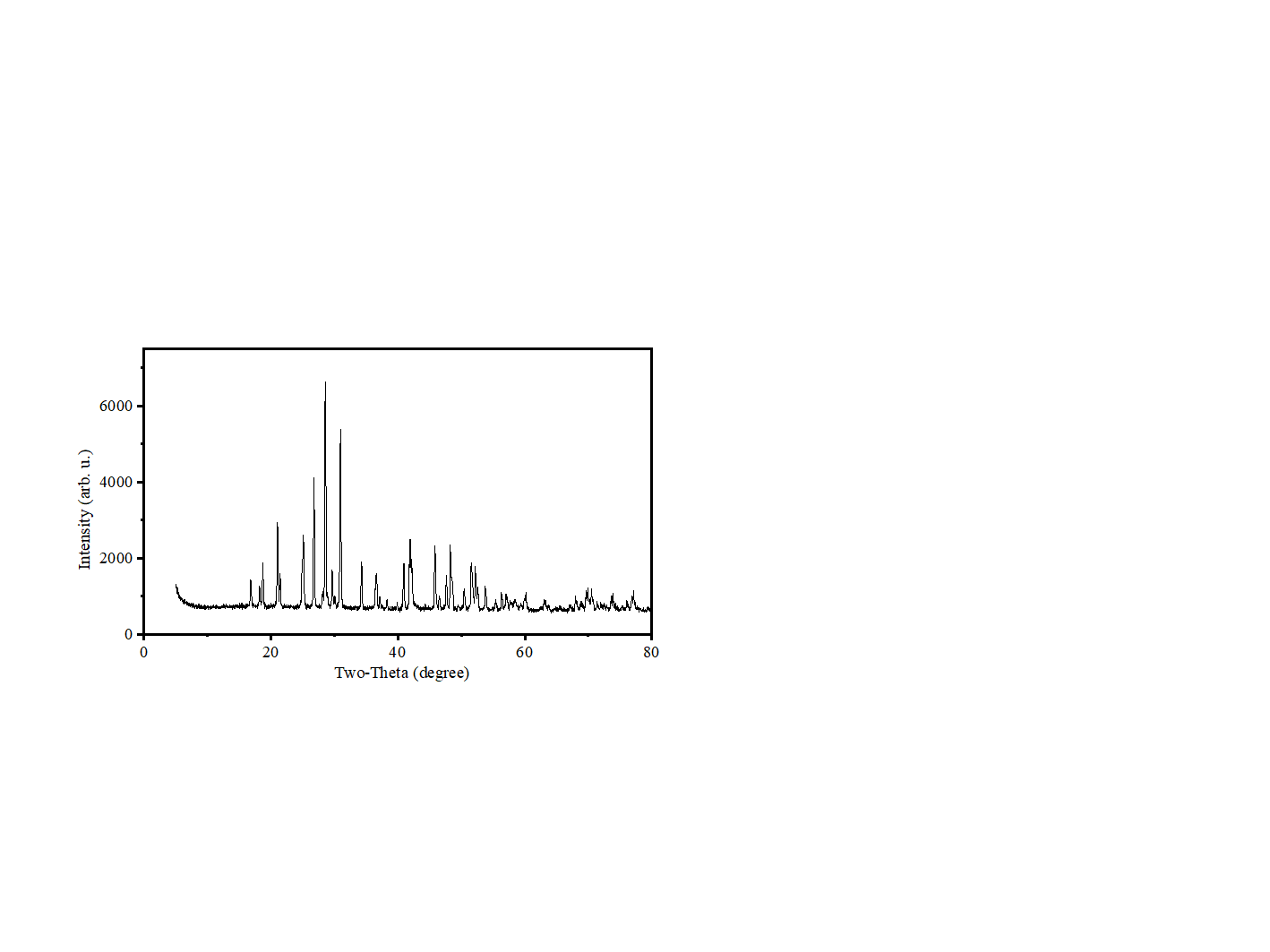


Fig. S28. XRD spectra of LaPO_4_: Tb^3+^, Ce^3+^& AlF_3_ samples sintered at 300 ℃


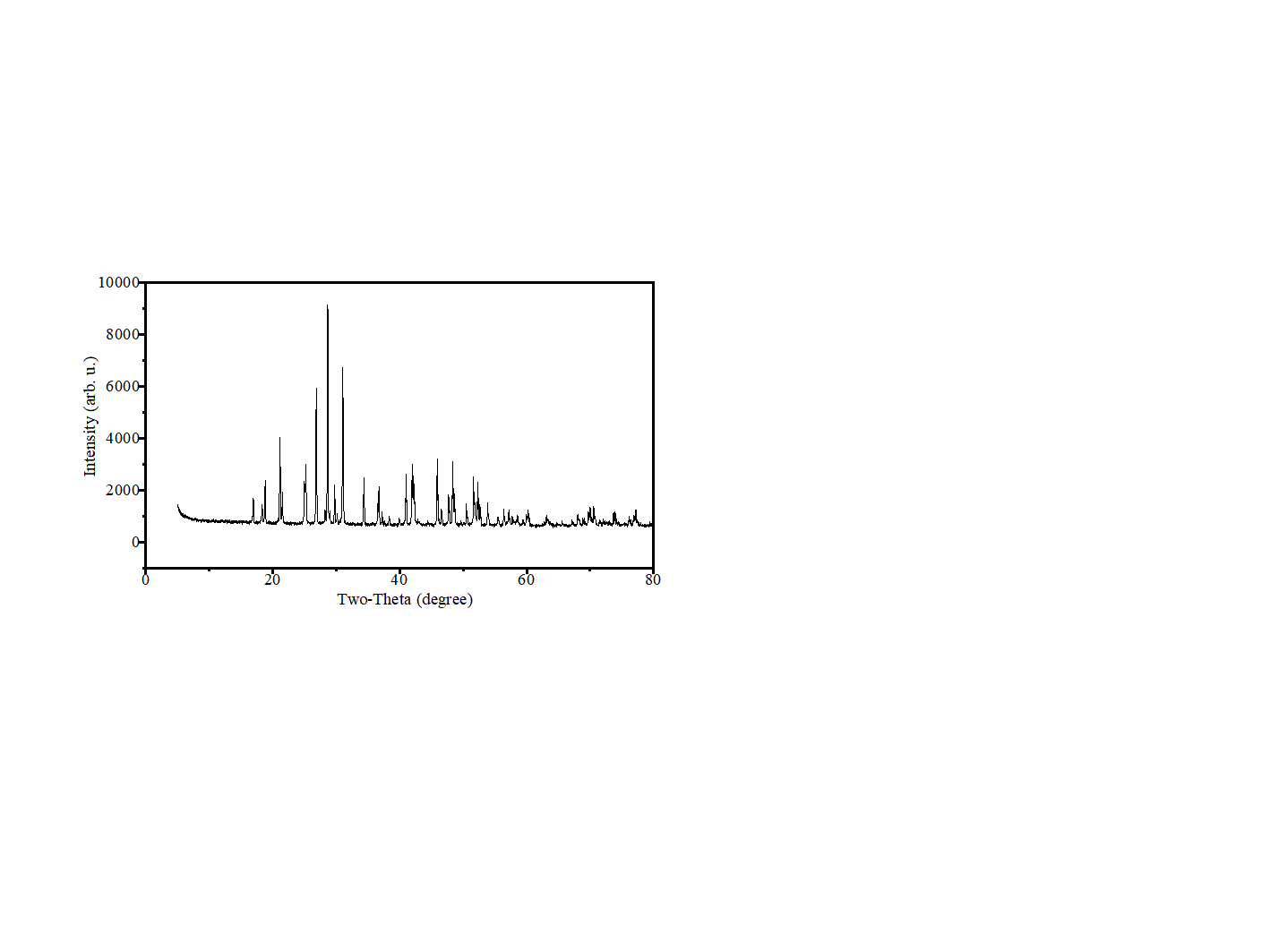


Fig. S29. XRD spectra of LaPO_4_: Tb^3+^, Ce^3+^& AlF_3_ samples sintered at 400 ℃


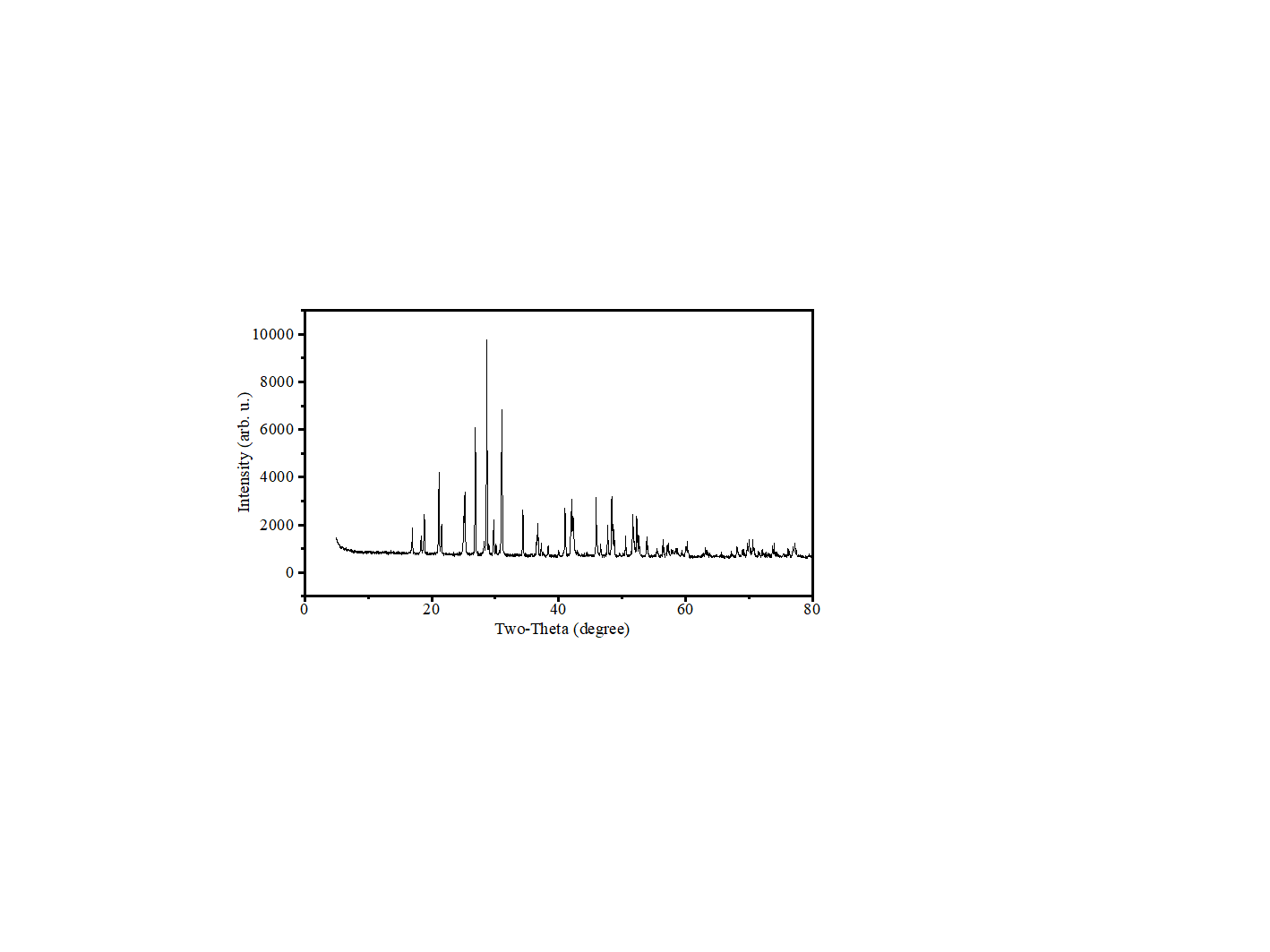


Fig. S30. XRD spectra of LaPO_4_: Tb^3+^, Ce^3+^& AlF_3_ samples sintered at 500 ℃


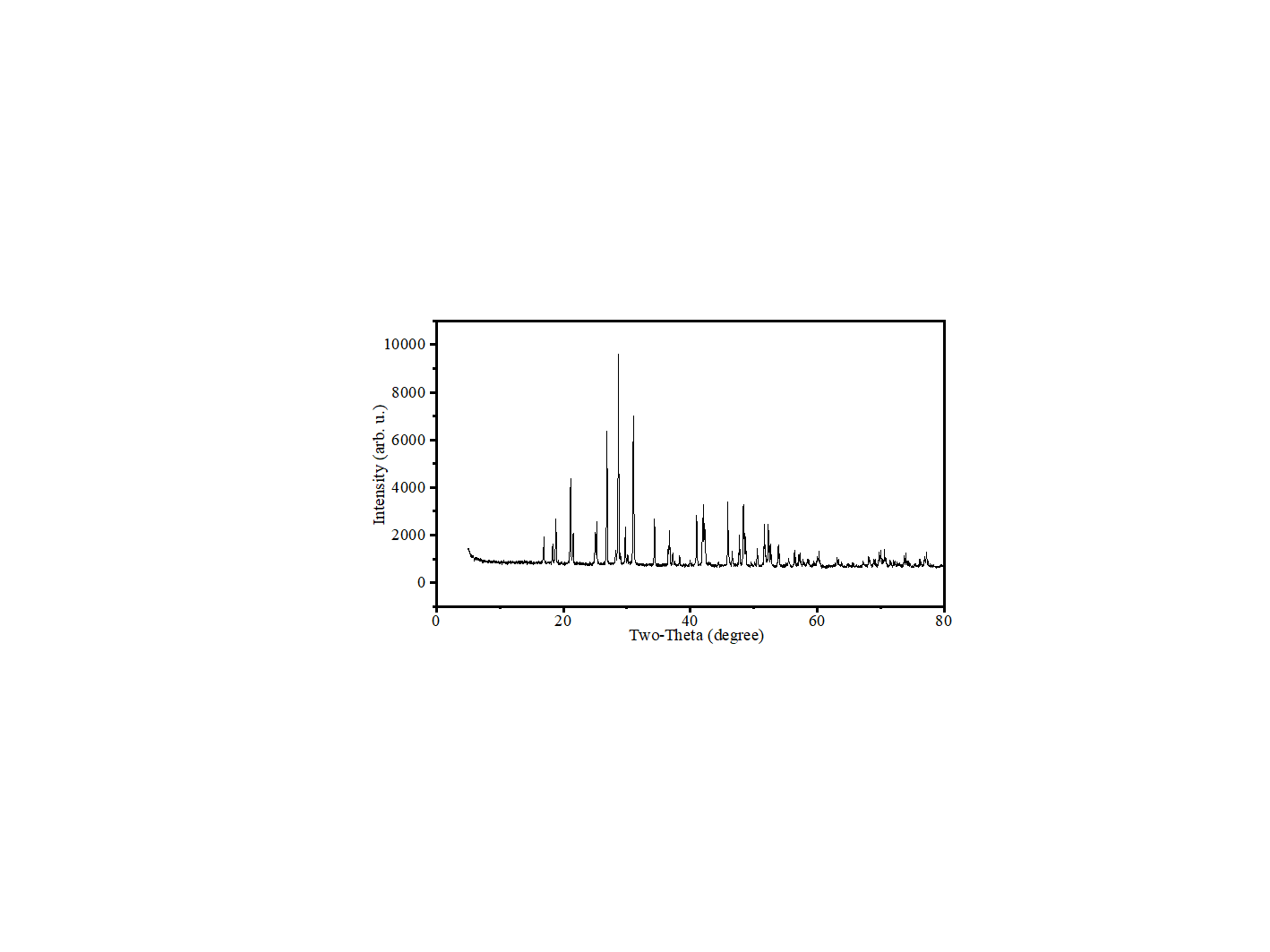


Fig. S31. XRD spectra of LaPO_4_: Tb^3+^, Ce^3+^& AlF_3_ samples sintered at 600 ℃

| Formula | LaPO_4_ |
| --- | --- |
| Crystal system | Monoclinic |
| Space group | P 21/n |
| Lattice parameters |  |
| a(Å) | 6.8060 |
| b(Å) | 7.0356 |
| c(Å) | 6.4826 |
| α°=r° | 90 |
| β° | 103.4382 |
| Cell volume(Å^3^ ) | 301.914 |
| T/K | 289 |
| Diffractometer | Rigaku D/Max-2400 |
| Radiation/Å | Cu-Ka (λ= 1.5405) |
| Absorption correction | multi-scan |
| 2θ range°/ | 10-80 |
| Z | 4 |
| Calculated Density | 5.1452 g/cm^3^ |
| R-factors |  |
| R_wp_ | 0.046 |
| R_p_ | 0.036 |

Table S1. Crystallographic data of LaPO_4_

| **Space group** | | | P 21/n | | | | | |
| --- | --- | --- | --- | --- | --- | --- | --- | --- |
| **Cell** | | | a = 6.8060(14) b =7.0356(14) c = 6.4826(13) | | | | | |
| **Ion coordinates** | **x** | | **y** | | **z** | **Occupancy** | | **U_iso_** |
| La1 | 0.2816(0) | | 0.1603(0) | | 0.1007(0) | 1.000(0) | | 0.0(0) |
| P1 | 0.3044(0) | | 0.1636(0) | | 0.612(0) | 1.000(0) | | 0.0(0) |
| O1 | 0.2494(0) | | 0.0077(0) | | 0.4458(0) | 1.000(0) | | 0.0(0) |
| O2 | 0.3803(0) | | 0.3306(0) | | 0.497(0) | 1.000(0) | | 0.0(0) |
| O3 | 0.476(0) | | 0.1081(0) | | 0.8023(0) | 1.000(0) | | 0.0(0) |
| O4 | 0.1289(0) | | 0.215(0) | | 0.7104(0) | 1.000(0) | | 0.0(0) |
| **The bond lengths of X-O** | | | | | | | | |
| **Vector** | | **Length** | | **Vector** | | | **Length** | |
| La1_O1 | | 2.53642(4) | | La1_O3 | | | 3.15475(4) | |
| La1_O1 | | 2.46571(5) | | La1_O4 | | | 2.53135(5) | |
| La1_O2 | | 2.77214(5) | | La1_O4 | | | 2.46603(5) | |
| La1_O2 | | 2.58294(4) | | P1_O1 | | | 1.522020(20) | |
| La1_O2 | | 2.65750(6) | | P1_O2 | | | 1.543400(20) | |
| La1_O3 | | 2.61093(4) | | P1_O3 | | | 1.539050(30) | |
| La1_O3 | | 2.49014(4) | | P1_O4 | | | 1.522670(30) | |

Table S2. Refined coordinates of all atoms, bond lengths of X-O and the unit cell parameters of the LaPO_4_ as determined by the GSAS program.

| Radius difference | | |
| --- | --- | --- |
| Element | La | P |
| Ce | 1.57% | 635.29% |
| Tb | 18.11% | 511.76% |

Table S3. Study on the Radius of Elements

| Element | radius | Electronegativity |
| --- | --- | --- |
| La | 1.27 | 1.249 |
| P | 0.17 | 2.139 |
| Ce | 1.25 | 1.264 |
| Tb | 1.04 | 1.313 |

Table S4. Study on the Electronegativity of Elements

| Poor electronegativity | | |
| --- | --- | --- |
| Element | La | P |
| Ce | 1.20% | 40.91% |
| Tb | 5.12% | 38.62% |

Table S5. Study on the Electronegativity of Elements

**Supplementary Note 1**

Generally, PDMS is an isotropic polymer with uniform surface potential distribution. In theory, single-crystal phosphors are anisotropic, and their surface potential is strongly dependent on crystal facets, namely certain facets tend to carry negative charges while others carry positive charges. Although each crystal facet can be electrically charged, the opposite charges offset each other, leading the overall macroscopic average potential close to zero. At present, macroscopic surface potential measured by electrometers is the most common testing method. For isotropic organic polymers such as PDMS, the integrated macroscopic potential presents a relatively high absolute value. In contrast, for polycrystalline inorganic phosphors, potentials with opposite signs from different particles and crystal facets neutralize one another, resulting in nearly neutral macroscopic electrical properties.

The only ideal strategy to objectively evaluate the contact electrification capability of inorganic crystals is to prepare single crystals with identical surface atomic termination, so as to confirm the definite surface potential of specific (hkl) crystal planes dominated by designated atoms. Apparently, such rigorous experimental conditions are extremely difficult to achieve. Accordingly, our analysis is mainly carried out from the theoretical perspective.

Work function comparison is currently the most widely adopted criterion for judging electron transfer direction. Our calculation results show that the work function of PDMS (4.58 eV) is lower than that of the phosphor (9.29 eV). According to the contact electrification theory, electrons tend to migrate from materials with lower work function (PDMS) to those with higher work function (phosphor) to achieve Fermi level equilibrium. Therefore, in the LaPO_4_/PDMS composite system, electrons transfer from PDMS to the phosphor surface. PDMS loses electrons and becomes positively charged, while the phosphor gains electrons and turns negatively charged. The built-in electric field formed accordingly provides essential excitation conditions for mechanoluminescence emission.

**Supplementary Note 2**

1. Distinct excitation energy. In this work, photoluminescence emission spectra are mainly acquired under excitation at 276 nm for selective excitation of Tb^3+^ ions. As displayed in Figure 2, the excitation spectrum of LaPO_4_:Tb^3+^,Ce^3+^ reaches its maximum at 276 nm. Hence, a narrow-band light source at 276 nm is adopted to obtain the standard atmospheric-pressure emission spectrum of Tb^3+^. In contrast, contact electrification serves as a broad-spectrum excitation source with a wide energy range of 3-5 eV as reported in our previous studies ^[1]^. Moreover, relevant literatures also prove that contact electrification can even emit X-rays ^[2,3]^. Apparently, different excitation sources tend to produce emission spectra with subtle discrepancies.

2. Different external pressure conditions. Conventional photoluminescence excitation and emission spectra are generally measured under atmospheric pressure, while ML spectra are collected under local high pressure. High pressure can slightly alter the lattice environment and energy levels of luminescent centers, further causing spectral broadening and peak shift, which has been widely verified in previous reports ^[4–7]^.

In addition, testing equipment difference remains the dominant factor for spectral divergence between photoluminescence and ML. ML is transient and faint, so its signals are collected by CCD cameras with relatively large slits, resulting in broadened emission peaks. By comparison, steady-state photoluminescence with high luminous intensity is tested via wavelength scanning using a fluorescence spectrometer with small slits to acquire high-precision spectral curves. Overall, the accuracy of ML spectra is inferior to that of photoluminescence spectra.

According to your significant suggestion, we have elaborated the above plausible mechanisms in the main text and supporting information revised manuscript.


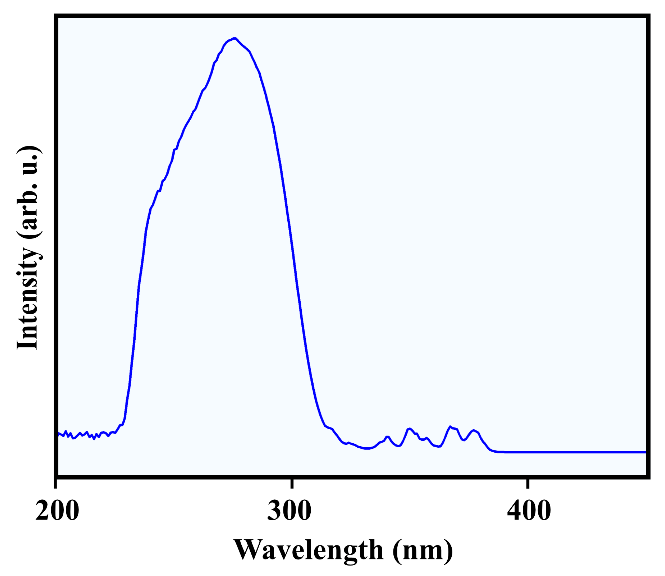


**Fig. S32.** Photoluminescence excitation spectrum of LaPO_4_:Tb^3+^,Ce^3+^ phosphor

**Supplementary Note 3**

we have supplemented relevant mechanical tests on pure PDMS elastomer and LaPO_4_: Tb^3+^, Ce^3+^@AlF_3_/PDMS composites, including tensile strength, elongation at break, elastic modulus and cyclic tensile fatigue property. As shown in the stress-strain curves in Fig. S33, pure PDMS possesses a breaking strength of 1.08 MPa, elongation at break of 0.92 and elastic modulus of 1.28 MPa. After incorporating phosphor and AlF_3_ fillers, the breaking strength and elastic modulus of the composite increase to 1.37 MPa and 1.47 MPa respectively, while the elongation at break slightly decreases to 0.89.

The test results reveal that the introduction of LaPO_4_: Tb^3+^, Ce^3+^ phosphors and AlF_3_ can moderately enhance the mechanical strength and rigidity of PDMS matrix, without obvious deterioration in ductility.


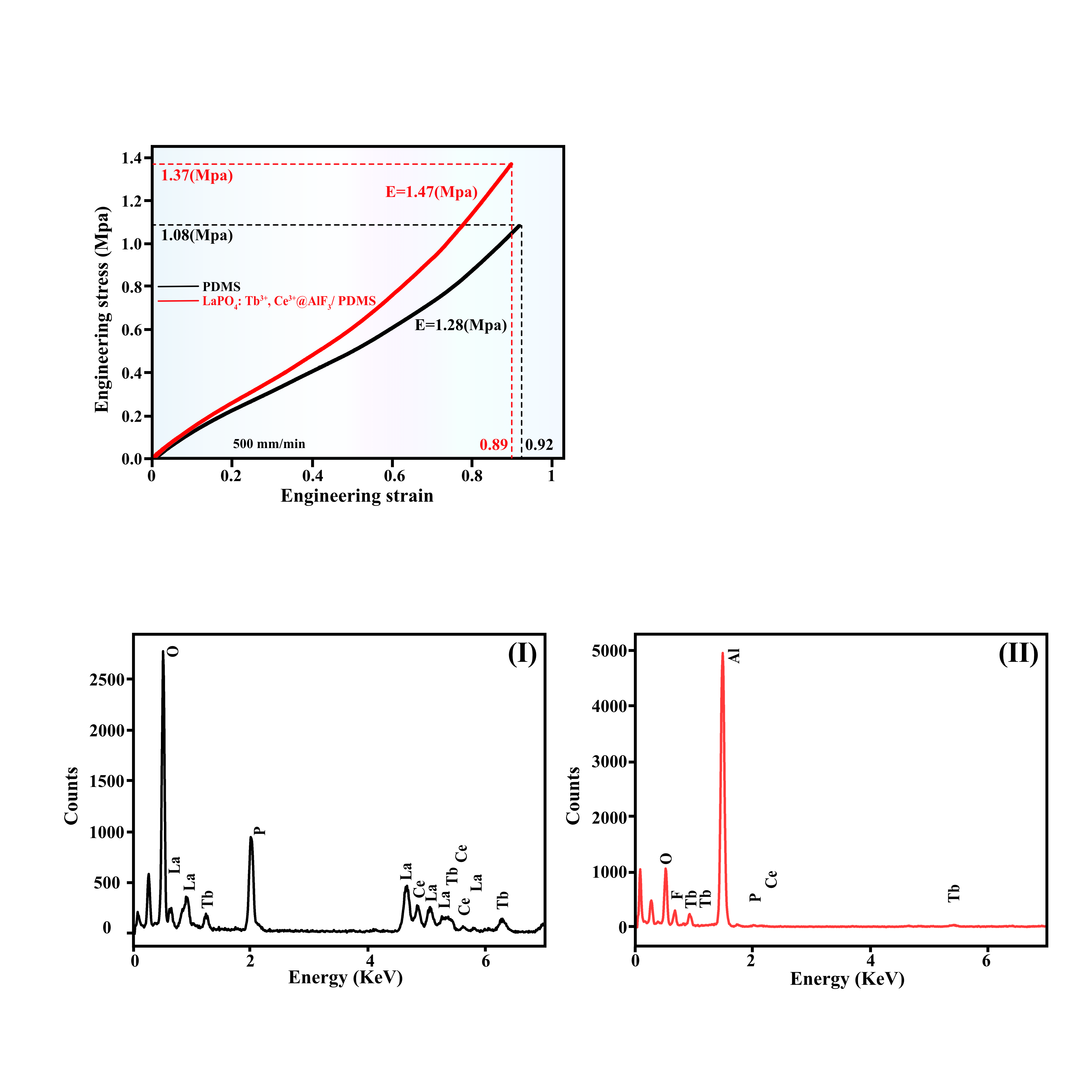


**Fig. S33.** Stress-strain curves of pure PDMS elastomer and LaPO_4_: Tb^3+^, Ce^3+^@AlF_3_/PDMS composite material

Furthermore, we have carried out 20 consecutive cyclic tensile tests on pure PDMS elastomer and LaPO_4_:Tb^3+^, Ce^3+^/PDMS composite material. As displayed in Fig. S34, no obvious variation in mechanical parameters or structural damage is observed for both samples after 20 tensile cycles, and their mechanical properties remain stable throughout the test. This result demonstrates that the LaPO_4_: Tb^3+^, Ce^3+^/PDMS composite possesses favorable mechanical stability and fatigue resistance, which can meet the practical application requirements of self-recoverable ML materials.


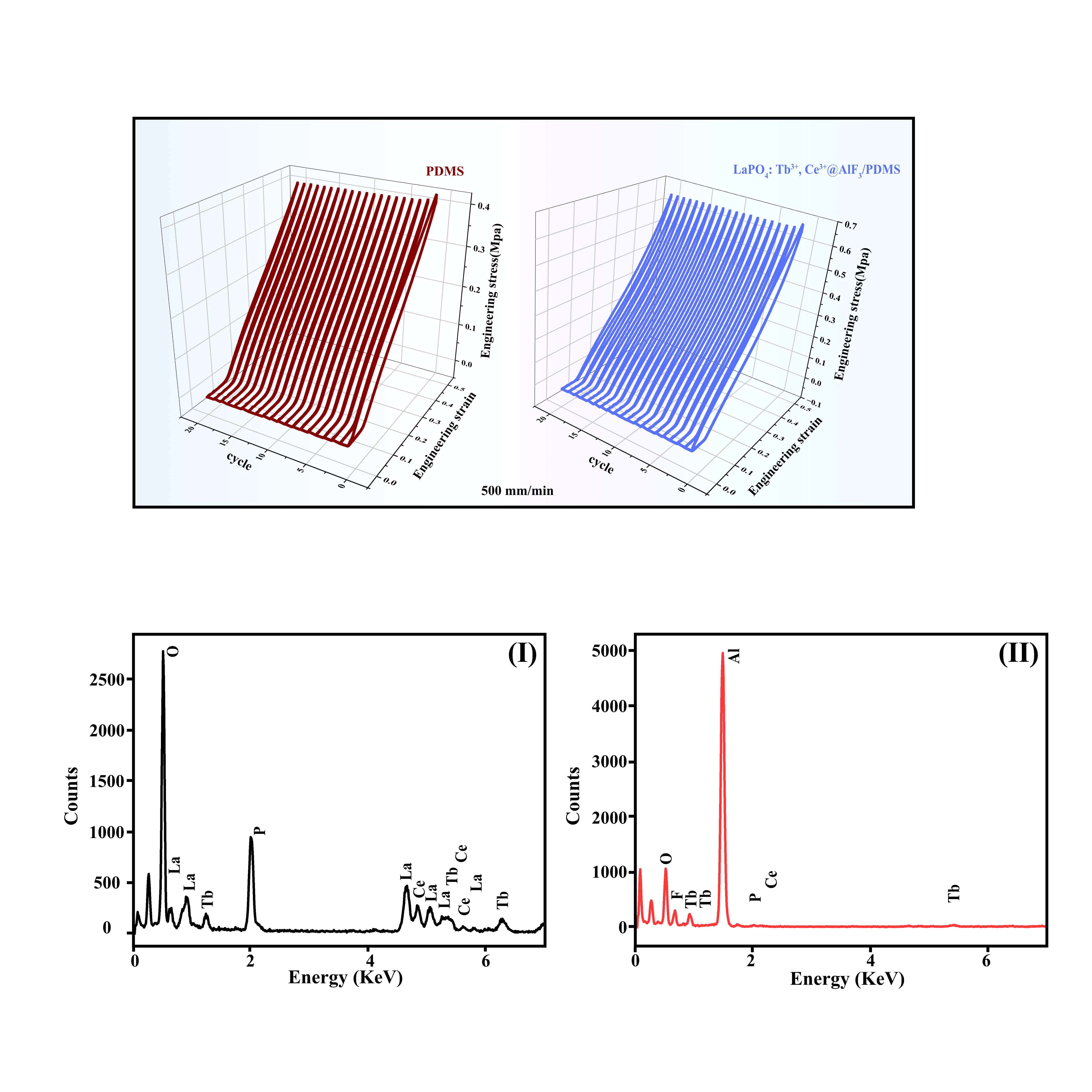


**Fig. S34.** Cyclic tensile stress-strain curves of LaPO_4_: Tb^3+^, Ce^3+^@AlF_3_/PDMS composite

**References**

[1] W. Wang, Z. Wang, J. Zhang, J. Zhou, W. Dong, Y. Wang, “Contact electrification induced mechanoluminescence ” *Nano Energy* 94 (2022): 106920.

[2] K. Sanderson, “Sticky tape generates X-rays *Nature”* (2008).

[3] D. Li, C. Xu, Y. Liao, W. Cai, Y. Zhu, Z. L. Wang, “Interface inter-atomic electron-transition induced photon emission in contact-electrification” *Science Advances* 7 (2021): eabj0349.

[4] J. Xue, M. Runowski, K. Soler-Carracedo, P. Woźny, A. Muñoz, L. Luo, K. Chen, Y. Huang, V. Lavín, P. Du, “Super-Sensitive Multi-Modal Optical Manometer Based on Huge Pressure-Induced Spectral Red-Shift and Broadening of Mn^2+^Emission Band in Green-Emitting Zn_2_GeO_4_ Phosphors*” Advanced Optical Materials* 13 (2025): 2402399.

[5] Q. Zeng, M. Runowski, J. Xue, L. Luo, L. Marciniak, V. Lavín, P. Du, “Pressure-Induced Remarkable Spectral Red-Shift in Mn^2+^-Activated NaY_9_(SiO_4_)_6_O_2_ Red-Emitting Phosphors for High-Sensitive Optical Manometry” *Advanced Science* 11 (2024): 2308221.

[6] Z. Wang, F. Wang, in *Luminescence - OLED Technology and Applications*, IntechOpen **2018**.

[7] H. Wang, T. Zhao, M. Li, J. Li, K. Liu, S. Peng, X. Liu, B. Zhao, Y. Chen, J. An, X. Chen, S. Jiang, C. Lin, W. Yang, “ Oscillatory mechanoluminescence of Mn^2+^-doped SrZnOS in dynamic response to rapid compression” *Nature Communications* 16 (2025): 548.
